# Chorioamnionitis Is a Risk Factor for Intraventricular Hemorrhage in Preterm Infants: A Systematic Review and Meta-Analysis

**DOI:** 10.3389/fphys.2018.01253

**Published:** 2018-09-11

**Authors:** Eduardo Villamor-Martinez, Monica Fumagalli, Owais Mohammed Rahim, Sofia Passera, Giacomo Cavallaro, Pieter Degraeuwe, Fabio Mosca, Eduardo Villamor

**Affiliations:** ^1^Department of Pediatrics, School for Oncology and Developmental Biology (GROW), Maastricht University Medical Center, Maastricht, Netherlands; ^2^Neonatal Intensive Care Unit, Department of Clinical Sciences and Community Health, Fondazione IRCCS Cà Granda Ospedale Maggiore Policlinico, Università degli Studi di Milano, Milan, Italy

**Keywords:** chorioamnionitis, intraventricular hemorrhage, very preterm infant, systematic review, meta-analysis

## Abstract

Although chorioamnionitis (CA) is a well-known risk factor for white matter disease of prematurity, the association with intraventricular hemorrhage (IVH) is controversial and has not been yet systematically reviewed. We performed a systematic review and meta-analysis of studies exploring the association between CA and IVH. A comprehensive literature search was conducted using PubMed/MEDLINE and EMBASE, from their inception to 1 July 2017. Studies were included if they examined preterm infants and reported primary data that could be used to measure the association between exposure to CA and the presence of IVH. A random-effects model was used to calculate odds ratios (OR) and 95% confidence intervals (CI). We found 1,284 potentially relevant studies, of which 85 met the inclusion criteria (46,244 infants, 13,432 CA cases). Meta-analysis showed that CA exposure was significantly associated with all grades IVH (OR 1.88, 95% CI 1.61–2.19), with grades 1–2 IVH (OR 1.69, 95% CI 1.22–2.34), and with grades 3–4 IVH (OR 1.62, 95% CI 1.42–1.85). Both clinical and histological CA were associated with an increased risk for developing IVH in very preterm infants. In contrast, the presence of funisitis did not increase IVH risk when compared to CA in the absence of funisitis (OR 1.22, 95% CI 0.89–1.67). Further meta-analyses confirmed earlier findings that CA-exposed infants have significantly lower gestational age (GA; mean difference [MD] −1.20 weeks) and lower birth weight (BW; MD −55 g) than the infants not exposed to CA. However, meta-regression and subgroup analysis could not demonstrate an association between the lower GA and BW and the risk of IVH in the CA-exposed infants. In conclusion, our data show that CA is a risk factor for IVH, but also a risk factor for greater prematurity and more clinical instability. In contrast to other complications of prematurity, such as patent ductus arteriosus, retinopathy of prematurity, or bronchopulmonary dysplasia, the effect of CA on IVH appears to be independent of CA as causative factor for very preterm birth.

## Introduction

Germinal matrix hemorrhage–intraventricular hemorrhage (GMH-IVH) is one of the most common complications of prematurity (Ballabh, 2010; Volpe, 2015; Inder et al., 2018). IVH typically initiates in the germinal matrix, which is a richly vascularized collection of neuronal-glial precursor cells in the developing brain and may disrupt the ependymal lining and extend into the lateral ventricle (Ballabh, 2010; Volpe, 2015; Inder et al., 2018). Severe IVH (grade 3–4) is associated with increased mortality as well as short- and long-term neurological morbidity, whilst the short-term and long-term outcomes of milder forms of IVH (grade 1–2) are less established, and they remain a significant research area (Volpe, 2015; Tortora et al., 2017; Inder et al., 2018).

As extensively reviewed by Inder et al. (2018) the pathogenesis of IVH is multifactorial and may involve intravascular, vascular, and extravascular factors. Intravascular factors relate to the regulation of blood flow, pressure, and volume in the microvascular bed of the germinal matrix as well as to platelet-capillary function and blood clotting capability (Inder et al., 2018). Vascular factors refer to the intrinsic fragility and vulnerability of germinal matrix blood vessels (Inder et al., 2018). Extravascular factors include the poor support of the extravascular space surrounding the germinal matrix capillaries, the postnatal decrease in extravascular tissue pressure, and an excessive fibrinolytic activity (Inder et al., 2018). As assessed by Inder et al. (2018) not all the pathogenetic factors are present in every IVH and the clinical circumstances determine which factors are most relevant in each infant. Among these clinical circumstances, very preterm birth, generally defined as birth before 32 completed weeks of gestation, is the most consistently associated with the development of IVH. However, a number of risk factors including, among others, absent antenatal corticosteroid (ACS) treatment, vaginal delivery, peri- and postnatal hypoxic-ischemic events, severe respiratory distress syndrome (RDS), pneumothorax, hypercapnia, hemodynamic disturbances (either systemic hypertension or hypotension), rapid volume expansion, decreased hematocrit, glucose and/or electrolyte disturbances, seizures, patent ductus arteriosus (PDA), thrombocytopenia, inherited thrombophilia, and infection may predispose to the development of IVH (Ballabh, 2010; Ramenghi et al., 2011; Volpe, 2015; Bermick et al., 2016; Romantsik et al., 2017; Inder et al., 2018; Poryo et al., 2018).

Several studies suggest that IVH is unequally distributed among the different leading causes of very preterm delivery (DiSalvo, 1998; Chevallier et al., 2017). An estimated 40% of very preterm births are associated with placental inflammation, which is often subclinical. This inflammation may be localized to the maternal placenta or membrane (chorioamnionitis) or may extend to the fetus, inducing an inflammatory response, which is evidenced by funisitis (Cornette, 2004; Gantert et al., 2010; Tita and Andrews, 2010; Thomas and Speer, 2011; Pugni et al., 2016; Jackson et al., 2017). Chorioamnionitis (CA) is not only a major risk factor for (very) preterm birth, but it is also considered a major risk factor for the morbidity and mortality associated with prematurity (Cornette, 2004; Gantert et al., 2010; Tita and Andrews, 2010; Thomas and Speer, 2011; Pugni et al., 2016; Jackson et al., 2017). The pathogenetic role of CA in the development of complications of prematurity, such as necrotizing enterocolitis (NEC), bronchopulmonary dysplasia (BPD), PDA, retinopathy of prematurity (ROP), or cerebral palsy has been addressed in several systematic reviews (Wu and Colford, 2000; Hartling et al., 2012; Been et al., 2013; Mitra et al., 2014; Park et al., 2015; Behbodi et al., 2016; Villamor-Martinez et al., 2018a). Although intrauterine inflammation is a well-known risk factor for white matter disease of prematurity (Strunk et al., 2014), the association with IVH is controversial and has not been yet systematically reviewed. Moreover, a consideration with any analysis of CA as a risk factor for preterm morbidity, is accounting for the role of GA, birth weight (BW) and other baseline characteristics which differ between CA-exposed and CA-unexposed infants (Hartling et al., 2012; Mitra et al., 2014; Behbodi et al., 2016; Villamor-Martinez et al., 2018a). With this in mind, we aimed to perform a systematic review and meta-analysis of studies exploring the association between CA and IVH, as well as the role of potential confounding factors.

## Methods

The methodology followed the same structure as earlier meta-analyses on CA and ROP (Villamor-Martinez et al., 2018a), and CA and PDA (Behbodi et al., 2016). We developed a protocol a priori, which specified the objectives, inclusion criteria, method for evaluating study quality, included outcomes and covariates, and statistical methodology. We report the study according to the guidelines for Preferred Reporting Items for Systematic Reviews and Meta-Analysis (PRISMA) (Moher et al., 2009).

### Sources and search strategy

We performed a comprehensive literature search in the PubMed/MEDLINE and EMBASE databases from their inception to July 1, 2017. The search strategy involved combining the following keywords in various ways: “chorioamnionitis,” “intrauterine infection,” “intrauterine inflammation,” “antenatal infection,” “antenatal inflammation,” “intraventricular hemorrhage,” “risk factors,” “outcome,” “cohort,” and “case-control.” No studies were excluded based on language. In addition, we used the following strategies to identify additional studies: review of reference lists of previous systematic reviews on CA, and of articles included in the present review, and the use of the “cited by” tool in Web of Science and Google Scholar.

### Study selection

We included studies which evaluated infants who were preterm (<37 weeks) or low BW (<2,500g), as well as studies which used stricter inclusion criteria. Studies were included if they reported primary data on the association between CA-exposure and IVH. We included studies which reported the rate of IVH in infants with and without CA, and studies which reported the rate of CA in infants with and without IVH. The results of the total search were screened independently by two reviewers (O.M.R, E.V.), in several rounds: first by title only, second by title and abstract, and thirdly by consulting the full text. The reviewers resolved discrepancies in inclusion through discussion and by consulting a third reviewer (P.D).

### Data extraction

Using a predetermined worksheet, two researchers (E.V.-M., O.M.R.) extracted data from the studies included. Another two investigators (P.D., E.V.) checked the extracted data for accuracy and completeness. We resolved discrepancies by discussion and through checking the primary report. The following data were extracted from each study: citation information, location of study, primary objective, criteria for inclusion/exclusion of infants, definitions used for CA and for IVH, infant baseline characteristics in the total group and the CA-exposed and CA-unexposed groups, and reported results on the outcomes of interest (including raw numbers, summary statistics and adjusted analyses on CA and IVH where available).

### Quality assessment

We used the Newcastle-Ottawa Scale (NOS) for cohort or case-control studies to assess the methodological quality of included studies. Three aspects of a study are evaluated by the NOS: selection, comparability and exposure/outcome, and these are scored individually and tallied up to a total of 9 points. Two researchers (E.V.-M. and E.V.) independently used the NOS to evaluate the quality of each study, and discrepancies were discussed and resolved by consensus.

### Statistical analysis

We combined and analyzed studies using comprehensive meta-analysis V 3.0 software (CMA, RRID:SCR_012779, Biostat Inc., Englewood, NJ, USA). We calculated the odds ratio (OR) and 95% confidence intervals (CI) for dichotomous outcomes from the data extracted from the studies. We calculated the mean difference (MD) and 95% CI for continuous outcomes. We used the method of Wan et al. (2014) to estimate the mean and standard deviation, when continuous variables were reported as median and range/interquartile range in studies. We used a random-effects model to calculate summary statistics, due to anticipated heterogeneity. This method accounts for both intra-study and inter-study variability.

A mixed-effects model was used for subgroup analyses (Borenstein et al., 2009a). This model is characterized by a random-effects model that combines studies within subgroups, and a fixed-effects model that combines subgroups together to create an overall effect. This model does not assume that study-to-study variance (tau-squared) is the same in all subgroups. We assessed statistical heterogeneity using the Cochran's Q statistic, which reflects the degree of variance, and the I^2^-statistic, which describes the proportion of observed variance that is due to variance in true effect sizes rather than sampling error (Borenstein et al., 2009b). Visual inspection of funnel plots and Egger's regression test were used to evaluate evidence of publication bias.

We used univariate random-effects meta-regression (method of moments) to evaluate covariates which may affect the effect size (Borenstein et al., 2009c). We defined the following covariates a priori as potential sources of variability: CA type (clinical or histological), funisitis, differences in GA and BW between the infants with and without CA, use of ACS, mode of delivery, rate of preeclampsia, rate of small for gestational age (SGA), rate of premature rupture of membranes (PROM), rate of RDS, rate of PDA, rate of early onset sepsis (EOS), rate of late onset sepsis (LOS) and mortality. We considered probability values under 0.05 (0.10 for heterogeneity) as statistically significant.

## Results

### Description of studies

**Figure 1:**
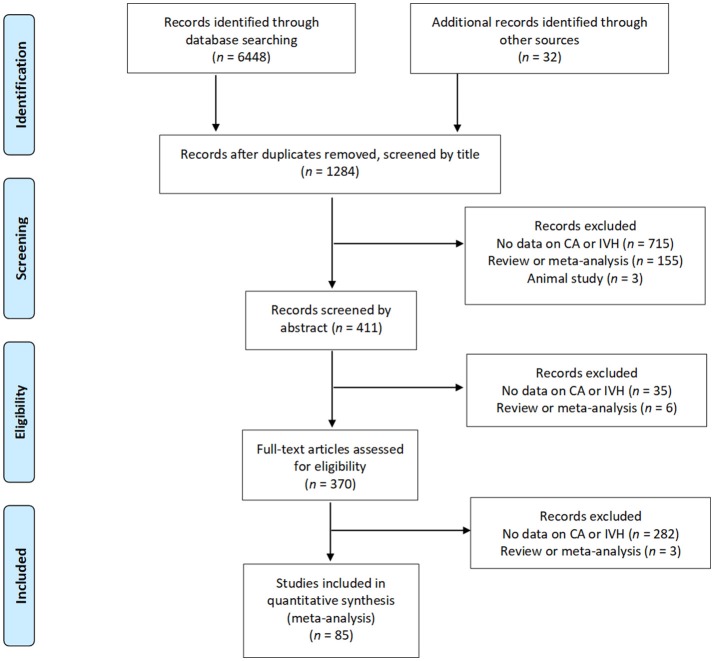
Flow diagram of search process (Moher et al., 2009).

After removing duplicates, we found 1,284 potentially relevant studies, of which 85 (Morales, 1987; Yoon et al., 1995; Gray et al., 1997; Alexander et al., 1998; Watterberg et al., 1999; Dexter et al., 2000; Elimian et al., 2000; Kosuge et al., 2000; Hitti et al., 2001; Suarez et al., 2001; González-Luis et al., 2002; Ohyama et al., 2002; Fung et al., 2003; Holcroft et al., 2003; Linder et al., 2003; Ogunyemi et al., 2003; Vergani et al., 2004; Dempsey et al., 2005; Lau et al., 2005; Osmanagaoglu et al., 2005; Polam et al., 2005; Sarkar et al., 2005; Babnik et al., 2006; Kaukola et al., 2006; Mehta et al., 2006; Richardson et al., 2006; Rocha et al., 2006; Yanowitz et al., 2006; Kirchner et al., 2007; Baumert et al., 2008; Mu et al., 2008; Zanardo et al., 2008; Been et al., 2009; Soraisham et al., 2009, 2013; Suppiej et al., 2009; Austeng et al., 2010; Botet et al., 2010; Kallankari et al., 2010; Lee et al., 2010, 2011; Mestan et al., 2010; Alfiero Bordigato et al., 2011; Barrera-Reyes et al., 2011; Hendson et al., 2011; Lim et al., 2011; Ryckman et al., 2011; Sato et al., 2011; Wirbelauer et al., 2011; Ahn et al., 2012; Klebermass-Schrehof et al., 2012; Perrone et al., 2012; Poralla et al., 2012; Rong et al., 2012; Vaihinger et al., 2012; van Vliet et al., 2012; Xu et al., 2012; Adén et al., 2013; Erdemir et al., 2013; Gawade et al., 2013; Logan et al., 2013; Nasef et al., 2013; Salas et al., 2013; Seliga-Siwecka and Kornacka, 2013; Trevisanuto et al., 2013; Tsiartas et al., 2013; Arayici et al., 2014; Ecevit et al., 2014; Gagliardi et al., 2014; García-Muñoz Rodrigo et al., 2014; Kidokoro et al., 2014; Liu et al., 2014; Pappas et al., 2014; Shankaran et al., 2014; Bry et al., 2015; Dalton et al., 2015; Kim et al., 2015; Oh et al., 2015, 2018; Smit et al., 2015; Yamada et al., 2015; Bermick et al., 2016; Lu et al., 2016; Miyazaki et al., 2016; Rodríguez-Trujillo et al., 2016) met the inclusion criteria. Figure 1 depicts the PRISMA flow diagram of the search. The included studies evaluated 46,244 infants, including 13,432 cases of CA. The characteristics of the included studies are summarized in Supplementary Table 1. Fifty-eight studies examined the outcomes of maternal CA and included IVH as one of the outcomes. Twenty-four studies evaluated risk factors for developing IVH and included maternal CA as of the risk factors. Five studies studied the association between CA and IVH as their primary outcome (De Felice et al., 2001; Sarkar et al., 2005; Babnik et al., 2006; Mehta et al., 2006; Zanardo et al., 2008). Fifty-four studies used a histological definition of CA and 24 studies used a clinical definition of CA. Only two studies (Hitti et al., 2001; Kirchner et al., 2007) examined microbiological CA and IVH. One study (Nasef et al., 2013) provided data on IVH and its association with histological and clinical CA separately. In four studies (Gray et al., 1997; Fung et al., 2003; Klebermass-Schrehof et al., 2012; Xu et al., 2012) infants were assigned to the CA group if they presented histological and/or clinical CA.

### Quality assessment

A summary of the quality assessment of each study using the NOS is shown in Supplementary Table 2. One study received a quality score of 5 points, 19 studies achieved a quality score of six points, 43 studies achieved a quality score of seven points, 11 studies achieved a quality score of eight, and 11 studies achieved the maximum score of 9 points. Studies were downgraded in quality for not adjusting the risk of IVH for confounders (*k* = 62), for not defining IVH clearly (*k* = 9), for only adjusting the risk of IVH for one confounding factor (*k* = 7), for not defining CA clearly (*k* = 6), and for losing a substantial portion of infants to follow-up (*k* = 4).

### Analysis based on unadjusted data

**Figure 2:**
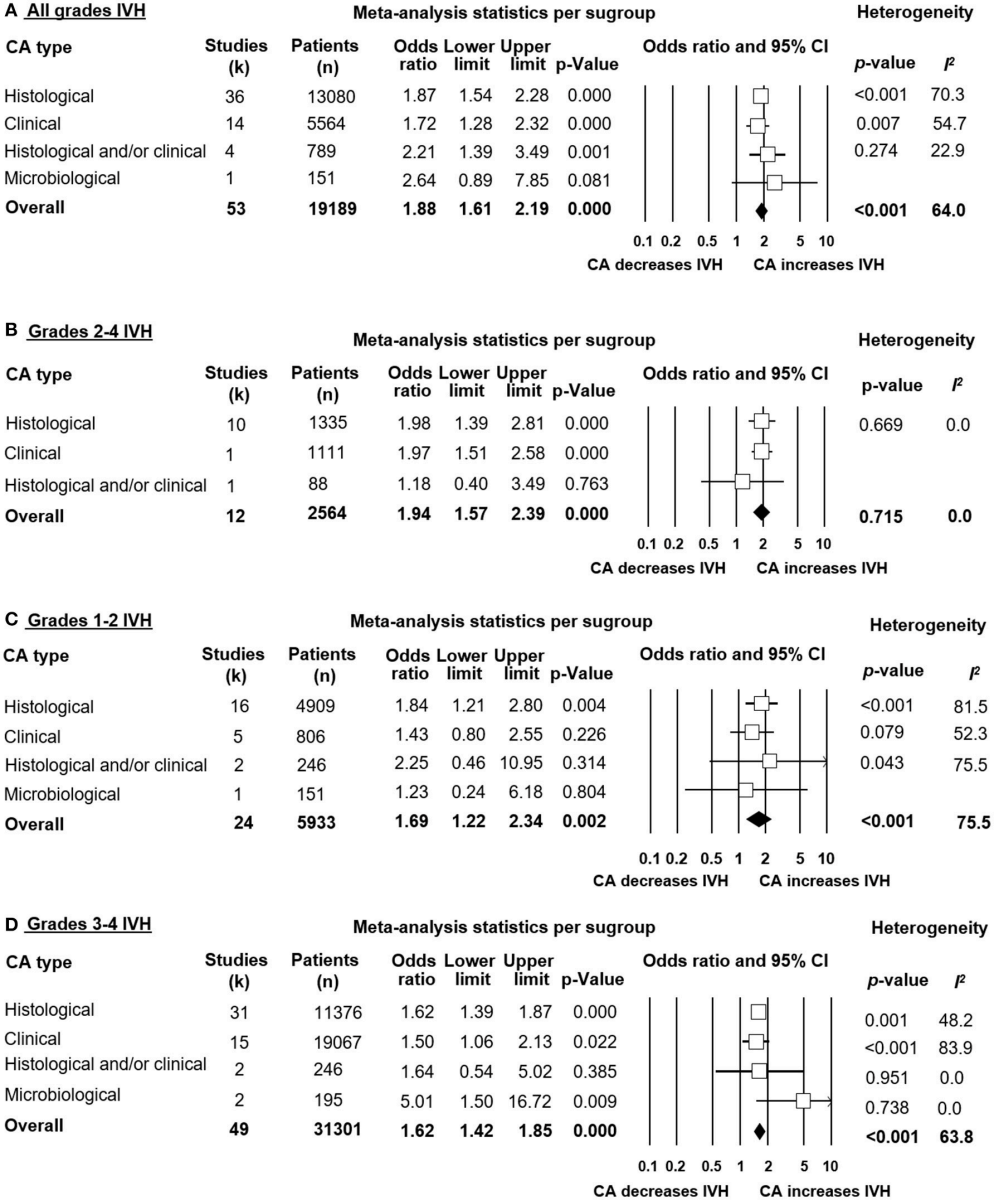
Meta-analyses of the association between chorioamnionitis (CA) and intraventricular hemorrhage (IVH), according to definition of IVH. CI, confidence interval. **(A)** CA and all grades IVH; **(B)** CA and grades 2-4 IVH; **(C)** CA and grades 1–2 IVH; **(D)** CA and grades 3–4 IVH.

**Figure 3:**
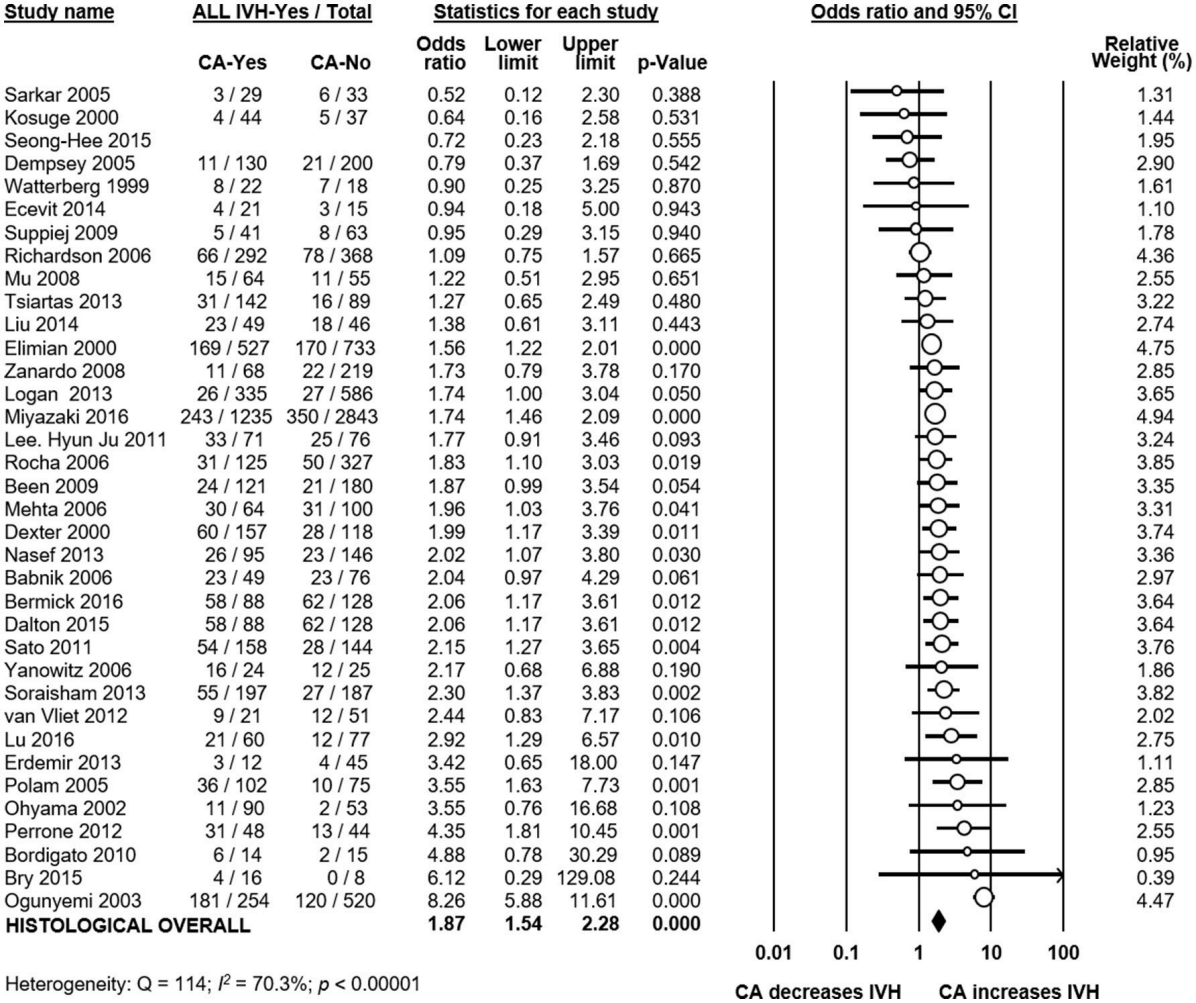
Meta-analysis of the association between histological chorioamnionitis (CA) and all grades intraventricular hemorrhage (IVH). CI, confidence interval.

**Figure 4:**
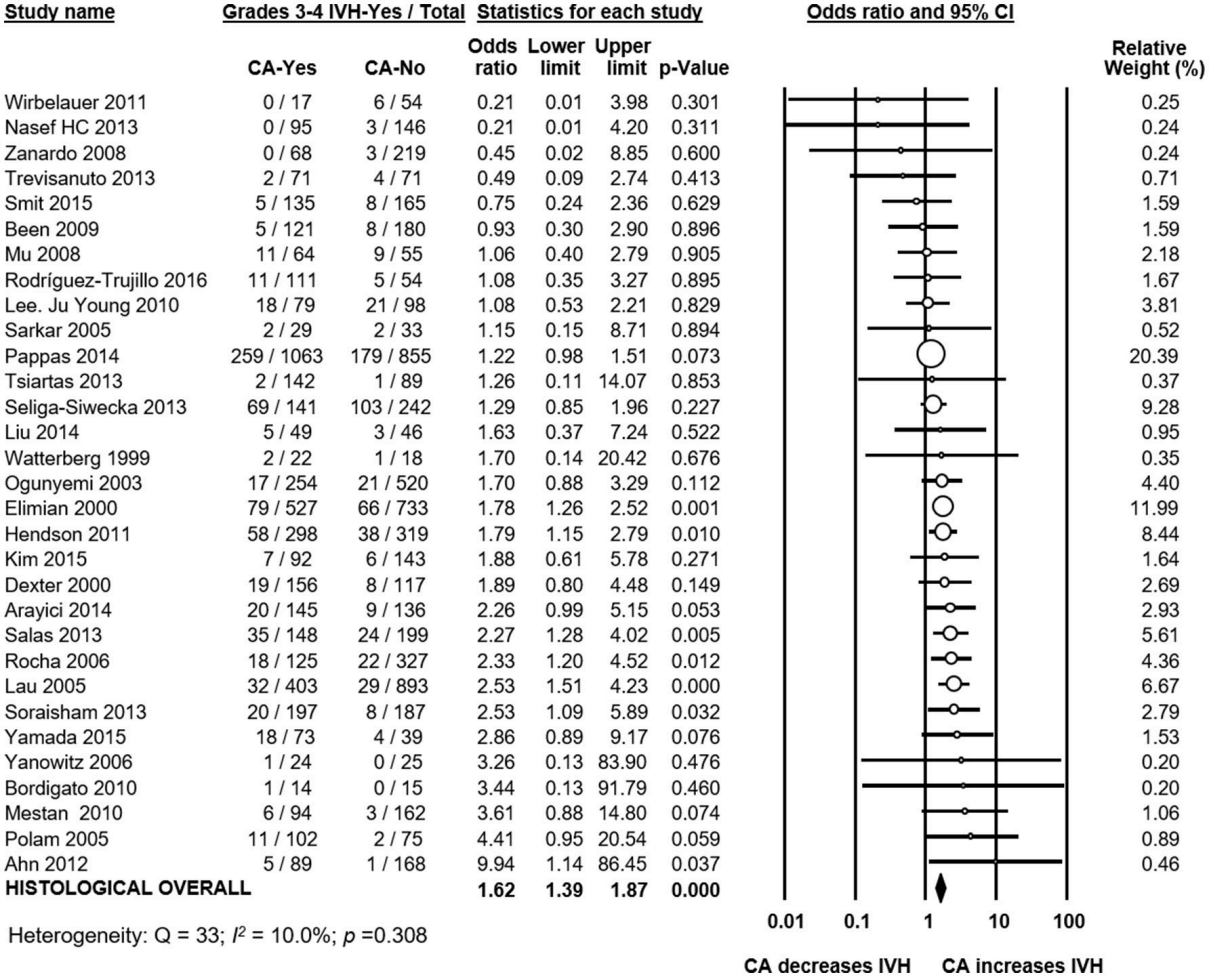
Meta-analysis of the association between histological chorioamnionitis (CA) and grades 3–4 intraventricular hemorrhage (IVH). CI, confidence interval.

**Figure 5:**
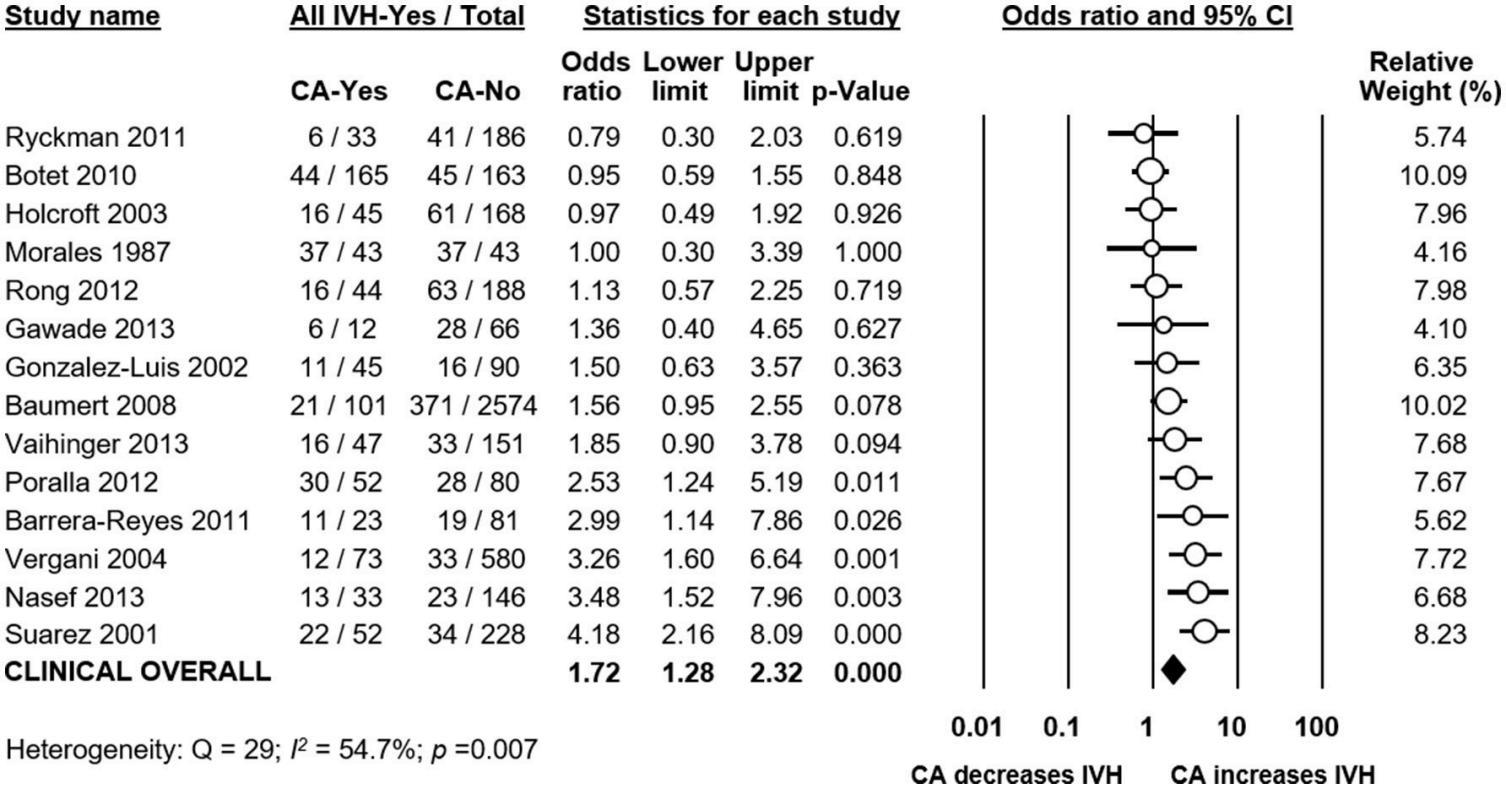
Meta-analysis of the association between clinical chorioamnionitis (CA) and all grades intraventricular hemorrhage (IVH). CI, confidence interval.

**Figure 6:**
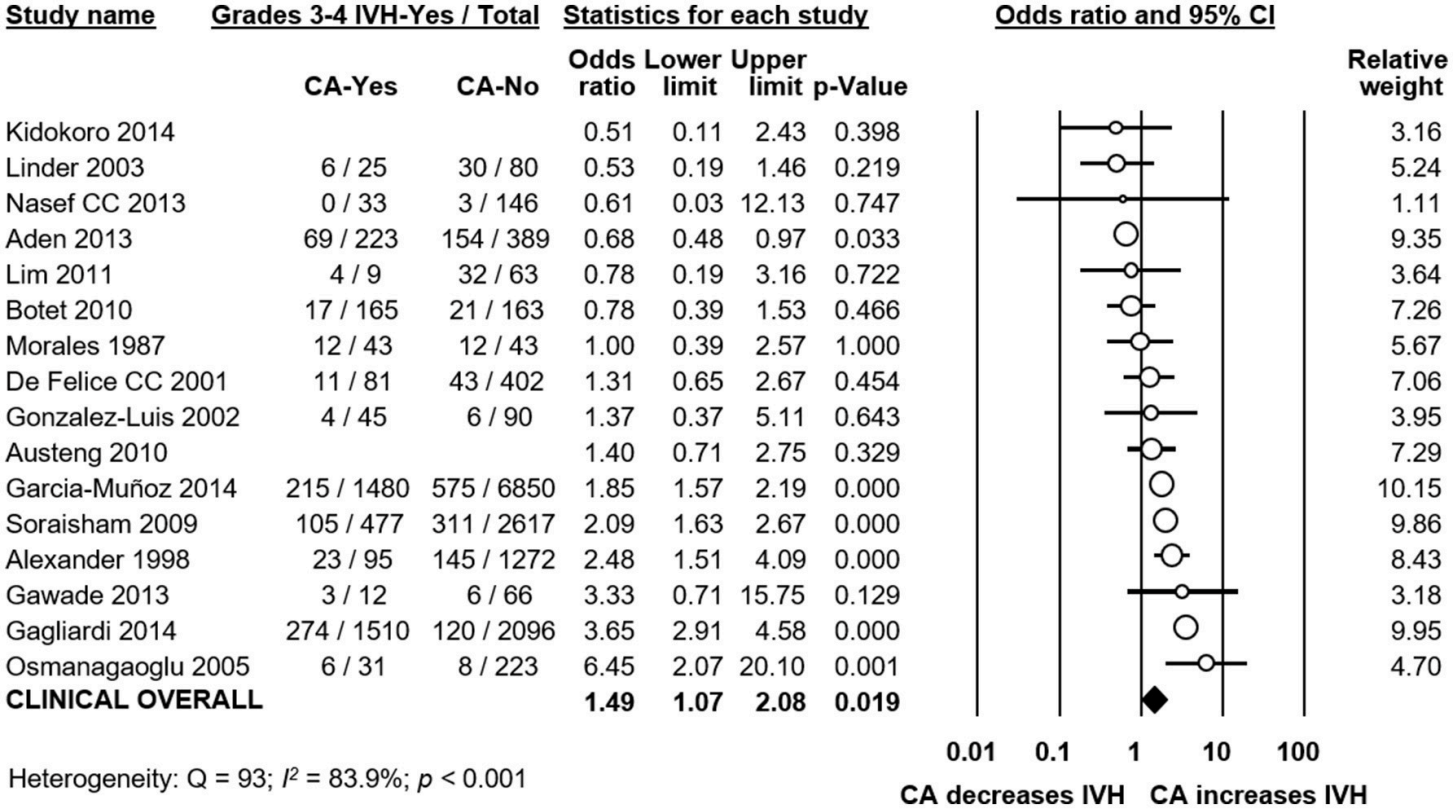
Meta-analysis of the association between clinical chorioamnionitis (CA) and grades 3–4 intraventricular hemorrhage (IVH). CI, confidence interval.

**Figure 7:**
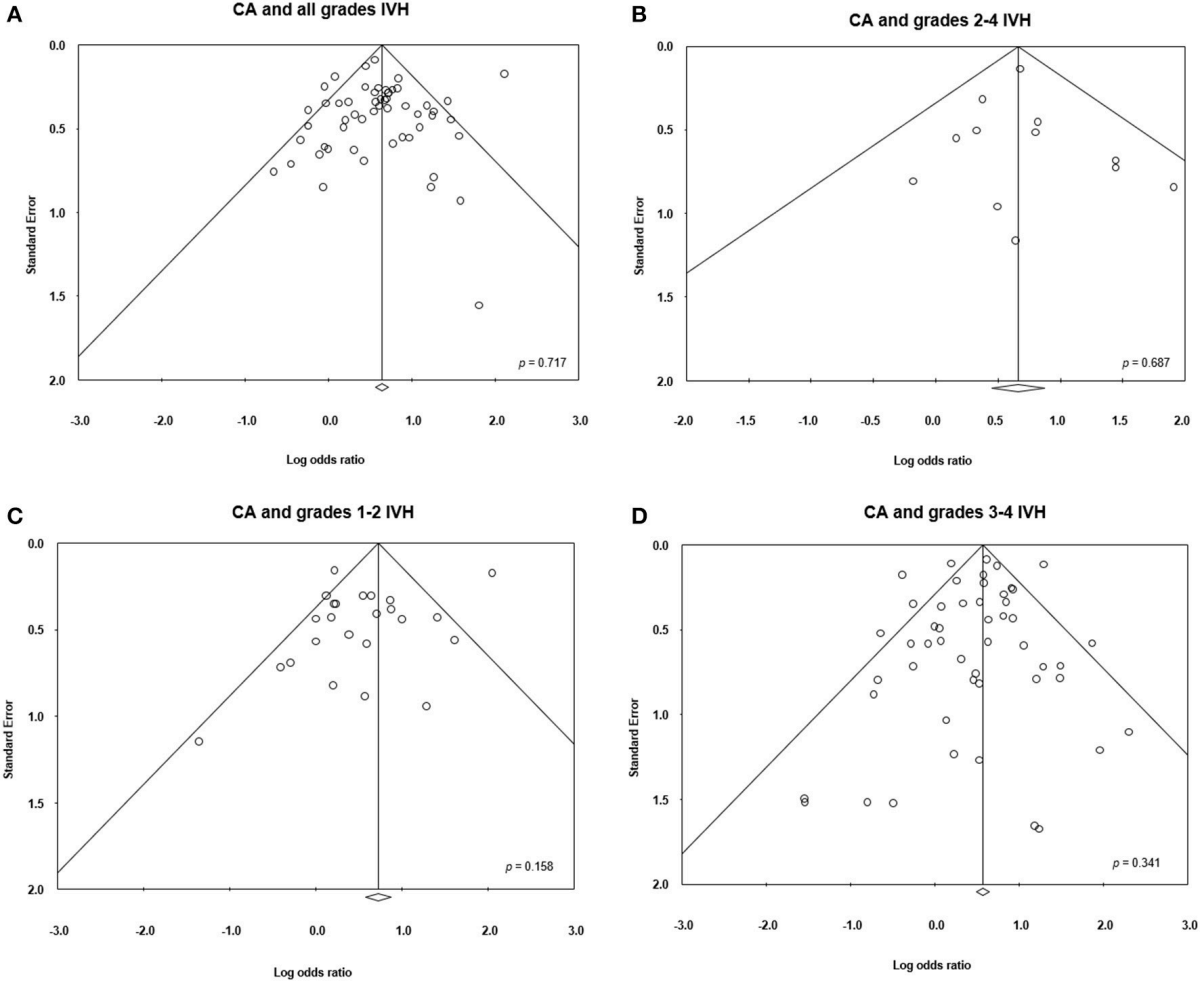
Funnel plots assessing publication bias for the association between chorioamnionitis (CA) and intraventricular hemorrhage (IVH).

Meta-analysis showed that CA exposure was significantly associated with all grades IVH (Figure 2A), with grades 2–4 IVH (Figure 2B), with grades 1–2 IVH (Figure 2C), and with grades 3–4 IVH (Figure 2D). When the type of CA was analyzed separately, histological CA remained significantly associated with all grades IVH (Figure 3), with grades 2–4 IVH (Figure 2B), with grades 1–2 IVH (Figure 2C), and with grades 3–4 IVH (Figure 4). Clinical CA was significantly associated with all grades IVH (Figure 5) and with grades 3–4 IVH (Figure 6), but not with grades 1–2 IVH (Figure 2C). There was only one study providing data on the association of clinical CA and IVH grades 2–4 (Figure 2B). We could not find significant evidence of publication bias through visual inspection of the funnel plot (Figure 7), or through Egger's regression test.

### Analysis of covariates

To confirm findings from earlier reports (Behbodi et al., 2016; Villamor-Martinez et al., 2018a) on the differences in baseline and clinical characteristics between infants with and without CA, we carried out further meta-analyses. Infants exposed to CA had significantly lower GA and BW, as shown in Table 1. Moreover, infants with CA had significantly higher rates of exposure to ACS, significantly higher rates of PROM, significantly higher rates of EOS, significantly higher rates of LOS, and significantly higher rates of PDA (Table 1). Infants with CA also had significantly lower rates of cesarean delivery, significantly lower rates of small for gestational age (SGA) and significantly lower rates of preeclampsia (Table 1).

**Table 1:** Meta-analysis of the association between chorioamnionitis and covariates.

**Meta-analysis**	**Chorioamnionitis**	***k***	**Effect size**	**95% CI**	***Z***	***p***	**Heterogeneity**		
							***Q***	***p***	***I***^2^
Gestational age (weeks)	Clinical	11	MD −0.73	−1.16 to −0.30	−3.35	0.001	140	<0.001	92.9
	Histological	42	MD −1.27	−1.49 to −1.05	−11.42	<0.001	495	<0.001	91.7
	Any type	56	MD −1.20	−1.40 to −1.00	−11.66	<0.001	839	<0.001	93.4
Birth weight (g)	Clinical	11	MD −29.14	−77.66 to 19.39	−1.18	0.239	107	<0.001	90.6
	Histological	41	MD −70.21	−96.71 to −43.72	−5.19	<0.001	362	<0.001	89.0
	Any type	55	MD −55.00	−74.89 to −35.12	−5.42	<0.001	474	<0.001	88.6
Antenatal corticosteroids	Clinical	5	OR 1.10	0.76 to 1.60	0.52	0.605	55	<0.001	92.8
	Histological	31	OR 1.20	1.01 to 1.42	2.03	0.043	95	<0.001	68.3
	Any type	38	OR 1.19	1.02 to 1.38	2.28	0.023	155	<0.001	76.1
Cesarean section	Clinical	8	OR 0.53	0.30 to 0.93	−2.23	0.026	316	<0.001	97.8
	Histological	28	OR 0.33	0.25 to 0.45	−7.32	<0.001	177	<0.001	84.8
	Any type	36	OR 0.37	0.29 to 0.47	−8.06	<0.001	495	<0.001	92.9
SGA	Histological	15	OR 0.33	0.23 to 0.49	−5.53	<0.001	79	<0.001	82.3
	Any type	16	OR 0.34	0.23 to 0.50	−5.64	<0.001	80	<0.001	81.2
Preeclampsia	Clinical	3	OR 0.16	0.09 to 0.29	−5.98	<0.001	2	0.369	<0.001
	Histological	23	OR 0.15	0.11 to 0.20	−13.09	<0.001	65	<0.001	66.2
	Any type	27	OR 0.15	0.12 to 0.20	−15.25	<0.001	69	<0.001	62.2
PROM	Clinical	3	OR 5.02	2.71 to 9.31	5.12	<0.001	2	<0.001	18
	Histological	27	OR 3.14	2.54 to 3.87	10.63	<0.001	149	<0.001	82.6
	Any type	30	OR 3.29	2.70 to 4.02	11.76	<0.001	155	<0.001	81.3
Male sex	Clinical	8	OR 1.10	0.80 to 1.53	0.58	0.560	80	<0.001	91.2
	Histological	35	OR 0.99	0.89 to 1.11	−0.15	0.881	91	<0.001	62.8
	Any type	46	OR 1.00	0.90 to 1.12	0.07	0.941	193	<0.001	76.7
Maternal diabetes	Any type	9	OR 0.81	0.65 to 1.01	−1.92	0.055	5	0.725	0.0
EOS	Clinical	7	OR 4.41	3.58 to 5.42	14.08	<0.001	9	0.197	30.3
	Histological	18	OR 2.62	1.88 to 3.65	5.68	<0.001	48	<0.001	64.9
	Any type	25	OR 3.81	3.20 to 4.54	14.96	<0.001	87	<0.001	72.4
LOS	Clinical	5	OR 1.41	1.10 to 1.81	2.68	0.007	17	0.002	76.8
	Histological	30	OR 1.53	1.27 to 1.84	4.45	<0.001	134	<0.001	78.3
	Any type	37	OR 1.55	1.34 to 1.80	5.82	<0.001	174	<0.001	79.3
PDA	Clinical	4	OR 1.30	1.04 to 1.64	2.27	0.023	7	0.062	59.0
	Histological	26	OR 1.41	1.15 to 1.72	3.35	0.001	144	<0.001	82.6
	Any type	31	OR 1.60	1.35 to 1.80	6.00	<0.001	195	<0.001	84.6
RDS	Clinical	3	OR 2.01	0.48 to 8.41	0.95	0.341	11	0.004	82.0
	Histological	15	OR 1.09	0.81 to 1.45	0.55	0.582	89	<0.001	84.3
	Any type	21	OR 1.00	0.78 to 1.29	0.01	0.990	149	<0.001	86.6

*CI, confidence interval; MD, mean difference; OR, odds ratio; SGA, small for gestational age; PROM, premature rupture of membranes; EOS, early-onset sepsis; LOS, late-onset sepsis; PDA, patent ductus arteriosus; RDS, respiratory distress syndrome*.

We carried out meta-regression analysis to determine the possible influence of GA and BW on the association between CA and IVH. As Table 2 shows, meta-regression did not find that differences in GA or BW had a significant effect on the association between CA and IVH.

**Table 2:** Random effects meta-regression of IVH risk in the chorioamnionitis group, and mean difference in gestational age and birth weight.

**IVH grade**	**Meta-regression**	***k***	**CC**	**95% CI**	***Z***	***p***	***R*^2^**
All grades IVH	Mean difference gestational age (per week)	35	−0.02	−0.19 to 0.16	−0.17	0.863	0.00
	Mean difference birth weight (per 100 g)	35	0.00	−0.001 to 0.001	0.07	0.942	0.00
Grades 1–2 IVH	Mean difference gestational age (per week)	20	0.05	−0.15 to 0.25	0.51	0.613	0.00
	Mean difference birth weight (per 100 g)	20	0.13	−0.07 to 0.33	1.27	0.203	0.54
Grades 3–4 IVH	Mean difference gestational age (per week)	37	−0.19	−0.43 to 0.04	−1.62	0.105	0.29
	Mean difference birth weight (per 100 g)	37	−0.10	−0.30 to 0.10	−1.01	0.312	0.00

*IVH, intraventricular hemorrhage; k, number of included studies; CC, coefficient; CI, confidence interval*.

To further analyze the effect of GA on the risk of IVH, we carried out subgroup analyses. We found that in the group of studies where the CA-group did not differ significantly (*p* > 0.05) in GA from the control group, CA was a risk factor for all grades IVH but not for grades 3–4 IVH (Table 3). We analyzed a subgroup of studies where the CA-group had a MD in GA of ≤0.5 weeks, and we found that CA was a risk factor for all grades IVH and for grades 3–4 IVH (Table 3). We also found that in studies where the CA-group had a MD in GA of less than 1 week, CA was a risk factor for all grades IVH and for grades 3–4 IVH (Table 3).

**Table 3:** Subgroup meta-analyses based on difference in gestational age (GA).

**Subgroup of studies**	**IVH definition**	***k***	**OR**	**95% CI**	***p***
Studies where CA-group did not differ significantly in GA from control (*p* > 0.05)	All grades IVH	15	1.59	1.20 to 2.10	0.001
	Grades 3–4 IVH	14	1.54	0.99 to 2.39	0.055
Studies where CA-group did differ significantly in GA from control (*p* < 0.05)	All grades IVH	20	1.71	1.46 to 2.01	<0.001
	Grades 3–4 IVH	23	1.90	1.56 to 2.33	0.000
Studies where CA-group had a MD in GA of ≤0.5 weeks compared to control	All grades IVH	11	1.66	1.25 to 2.22	<0.001
	Grades 3–4 IVH	13	1.36	1.03 to 1.80	0.028
Studies where CA-group had a MD in GA of >0.5 weeks compared to control	All grades IVH	24	1.68	1.43 to 1.98	<0.001
	Grades 3–4 IVH	23	1.99	1.64 to 2.41	<0.001
Studies where CA-group had a MD in GA of <1 weeks compared to control	All grades IVH	16	1.72	1.37 to 2.15	<0.001
	Grades 3–4 IVH	18	1.52	1.21 to 1.92	<0.001
Studies where CA-group had a MD in GA of ≥1 weeks compared to control	All grades IVH	19	1.65	1.37 to 1.97	<0.001
	Grades 3–4 IVH	18	2.00	1.60 to 2.50	<0.001

*CA, chorioamnionitis; GA, gestational age; IVH, intraventricular hemorrhage; k, number of studies included; CI, confidence interval; MD, mean difference*.

To evaluate the role of other prespecified covariates in the association between CA and IVH, we performed additional meta-regression analyses. Meta-regression could not find a significant difference in IVH risk between infants with clinical and infants with histological CA (Table 4). Meta-regression did find a significant association between the CA-associated risk of grades 3–4 IVH and the risk of preeclampsia (Supplementary Figure 1), mortality (Supplementary Figure 2), risk of LOS (Supplementary Figure 3) and risk of PDA (Supplementary Figure 4) Other meta-regressions could not find a significant association between the CA-associated risk of IVH and other covariates (Table 4).

**Table 4:** Random effects meta-regression of IVH risk in the chorioamnionitis group, and predefined covariates.

**IVH grade**	**Meta-regression**	***k***	**CC**	**95% CI**	***Z***	***P***	***R*^2^**
All grades IVH	Chorioamnionitis type (histological/clinical)	49	−0.08	−0.45 to 0.29	−0.42	0.673	0.00
	Funisitis (CA+F+ vs. CA+F–)	9	−0.12	−0.62 to 0.38	−0.49	0.627	0.00
	ACS (log OR)	25	0.01	−0.46 to 0.49	0.05	0.964	0.00
	Cesarean section (log OR)	22	−0.07	−0.47 to 0.32	−0.36	0.717	0.00
	Maternal age (MD)	17	−0.08	−0.24 to 0.07	−1.09	0.276	0.00
	SGA (log OR)	11	0.39	−0.09 to 0.88	1.59	0.111	0.54
	PROM (log OR)	20	−0.40	−1.06 to 0.26	−1.19	0.233	0.08
	Preeclampsia (log OR)	16	0.03	−0.40 to 0.45	0.13	0.897	0.00
	Mortality (log OR)	26	0.18	−0.15 to 0.50	1.07	0.285	0.07
	Early onset sepsis (log OR)	14	0.13	−0.47 to 0.73	0.42	0.672	0.00
	Late onset sepsis (log OR)	24	−0.04	−0.39 to 0.32	−0.19	0.846	0.00
	PDA (log OR)	22	0.13	−0.21 to 0.48	0.75	0.453	0.00
	RDS (log OR)	21	0.02	−0.22 to 0.27	0.22	0.827	0.00
Grades 3–4 IVH	Chorioamnionitis type (histological/clinical)	46	−0.07	−0.44 to 0.30	−0.37	0.708	0.00
	Funisitis (CA+F+ vs. CA+F–)	8	0.07	−0.29 to 0.44	0.40	0.691	0.00
	ACS (log OR)	28	−0.11	−0.60 to 0.38	0.42	0.672	0.00
	Cesarean section (log OR)	27	0.08	−0.09 to 0.26	0.92	0.358	0.06
	Maternal age (MD)	18	−0.01	−0.25 to 0.22	−0.11	0.911	0.00
	SGA (log OR)	12	0.24	−0.19 to 0.67	1.08	0.280	0.22
	PROM (log OR)	22	−0.09	−0.35 to 0.17	−0.65	0.515	0.00
	Preeclampsia (log OR)	18	0.41	0.20 to 0.63	3.74	0.0004	1.00
	Mortality (log OR)	30	0.42	0.17 to 0.67	3.33	0.001	0.58
	Early onset sepsis (log OR)	20	0.11	−0.32 to 0.54	0.51	0.613	0.00
	Late onset sepsis (log OR)	26	0.35	0.01 to 0.70	1.99	0.047	0.22
	PDA (log OR)	21	0.40	0.04 to 0.76	2.18	0.029	0.48
	RDS (log OR)	27	−0.06	−0.34 to 0.21	−0.49	0.627	0.14

*k, number of studies included; CA, chorioamnionitis; F, funisitis; CC, coefficient; CI, confidence interval; MD, mean difference; OR, odds ratio; ACS, antenatal corticosteroids; SGA, small for gestational age; PROM, premature rupture of membranes; PDA, patent ductus arteriosus; RDS, respiratory distress syndrome*.

### Analysis of funisitis

**Figure 8:**
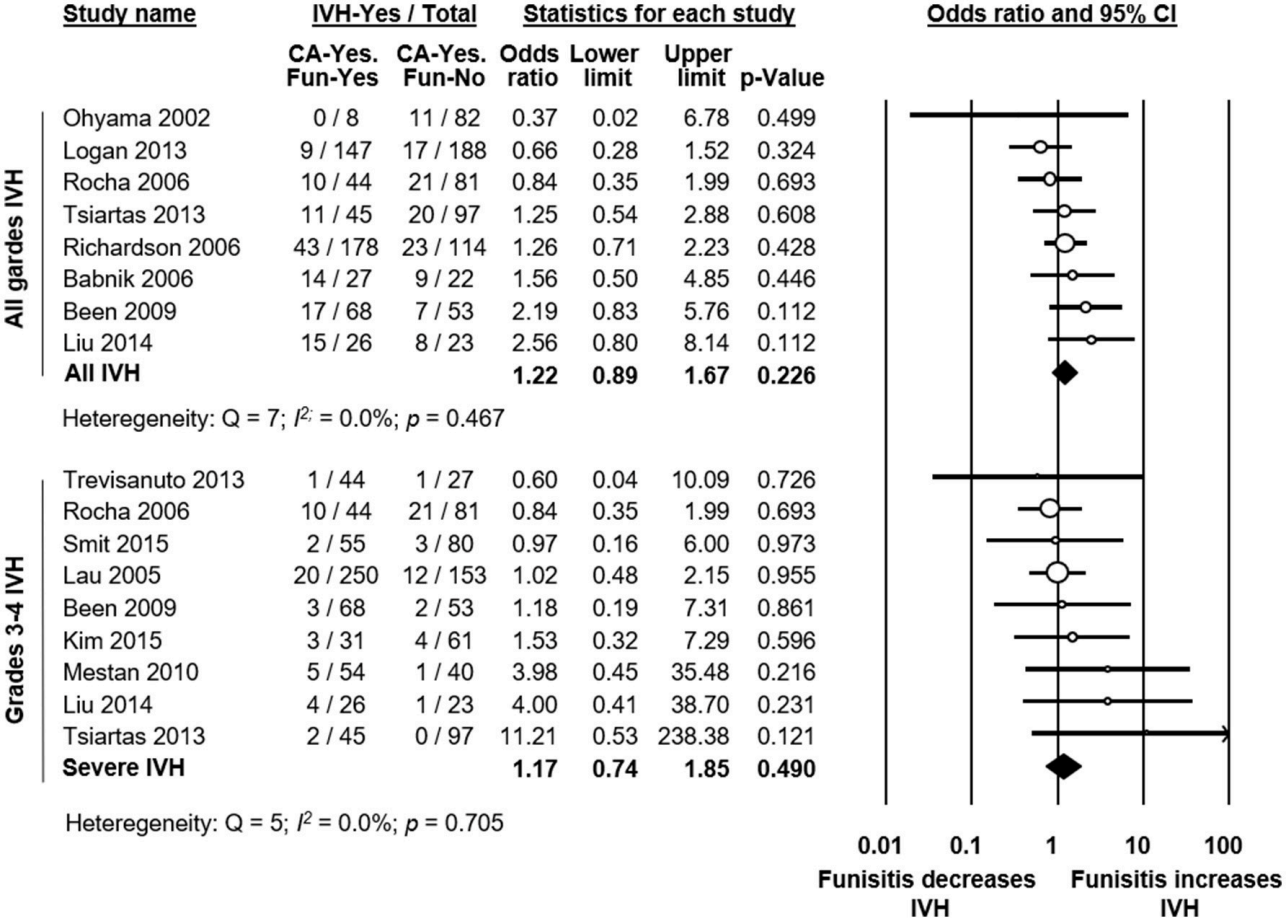
Meta-analysis of the association between funisitis and intraventricular hemorrhage (IVH). Fun, funisitis; CI, confidence interval.

To evaluate the role of funisitis (i.e., fetal inflammatory response) in the development of IVH, we carried out further meta-analyses. Thirteen studies reported on IVH (Ohyama et al., 2002; Lau et al., 2005; Babnik et al., 2006; Richardson et al., 2006; Rocha et al., 2006; Been et al., 2009; Mestan et al., 2010; Logan et al., 2013; Trevisanuto et al., 2013; Tsiartas et al., 2013; Liu et al., 2014; Kim et al., 2015; Smit et al., 2015) in infants with histological CA with or without funisitis. As shown in Figure 8, meta-analysis could not show a significant difference in IVH risk between infants with funisitis and infants with CA without funisitis (OR all grades IVH: 1.22, 95% CI 0.89 to 1.67; grades 3-4 IVH: 1.17, 95% CI 0.74 to 1.85). Using meta-regression, we also found no significant difference in IVH risk between infants with funisitis, and infants with CA without funisitis (Table 4).

### Analysis based on adjusted data

Thirteen studies adjusted the association between CA and the risk of IVH for confounding factors. As shown in Supplementary Tables 3, 4, studies adjusted for different covariates. Meta-analysis pooling this adjusted data found that CA was significantly associated with a higher risk of all grades IVH (OR 1.25, 95% CI 1.02–1.53, Supplementary Table 3). This association became non-significant when only analyzing studies which used a histological definition of CA (Supplementary Table 3). Meta-analysis of adjusted data also found a significant association between CA and grades 3–4 IVH (OR 1.22, 95% CI 1.04–1.43, Supplementary Table 4). This association became non-significant when grouping studies by clinical or histological CA definition (Supplementary Table 4).

## Discussion

The current systematic review and meta-analysis demonstrates that both clinical and histological CA are associated with an increased risk for developing IVH in very preterm infants. In contrast, the presence of funisitis did not increase IVH risk when compared to CA in the absence of funisitis. We found through additional meta-analyses that CA-exposed infants had significantly lower GA and BW than infants not exposed to CA. However, meta-regression and subgroup analysis could not demonstrate an association between the lower GA and BW and the risk of IVH in the CA-exposed infants. This suggests that the effects of CA on IVH risk might be at least partially independent on the role of CA as an etiological factor for very preterm birth.

The association between CA and increased risk of IVH is biologically and clinically plausible. IVH generally occurs within the three first days of life and affects the infants with higher hemodynamic and respiratory instability, frequently associated with extreme prematurity and/or severe perinatal infections (Mohamed and Aly, 2010; Inder et al., 2018). Therefore, the clinical circumstances around birth and during the first days of life are critical for the development of IVH. Our study confirms previous reports showing that these clinical circumstances are different in CA-exposed and CA–unexposed very preterm infants (Hartling et al., 2012; Behbodi et al., 2016; Villamor-Martinez et al., 2018a). Thus, CA-exposed infants were born 1.2 weeks earlier, they were 55g lighter at birth, and they were more frequently exposed to ACS, PROM, vaginal delivery, early and late onset sepsis, and PDA. As mentioned in the introduction, some of these factors may have affected IVH risk.

The degree of prematurity is the most important predisposing factor for the occurrence of IVH (Ballabh, 2010; Volpe, 2015; Inder et al., 2018), as well as for other complications of prematurity such as BPD, ROP, NEC, or PDA (Hartling et al., 2012; Been et al., 2013; Mitra et al., 2014; Behbodi et al., 2016; Villamor-Martinez et al., 2018a). Nevertheless, very preterm birth is always a pathological event and very preterm infants have a morbidity and mortality risk associated with whichever condition led to their early delivery (McElrath et al., 2008; Wilcox et al., 2011; Gagliardi et al., 2013; Barros et al., 2015). Therefore, CA may affect infant morbidity through inducing very preterm birth or through the deleterious effects of infection/inflammation. Interestingly, previous meta-analyses showed an association between the lower gestational age of the CA-exposed group and the CA-associated risk of BPD (Hartling et al., 2012), PDA (Behbodi et al., 2016), and ROP (Mitra et al., 2014; Villamor-Martinez et al., 2018a). In contrast, our meta-regression could not show that the difference in GA between CA-exposed and CA-unexposed infants significantly correlated with IVH risk. Moreover, we performed subgroup analyses in which we only included the studies showing small or no differences in GA between the CA-exposed and the control group and we observed that the significant IVH risk was maintained in this subgroup of studies. In contrast, this was not the case when the same subgroup analysis was performed for PDA (Behbodi et al., 2016) or ROP (Mitra et al., 2014; Villamor-Martinez et al., 2018a). Altogether this suggests that CA may increase complications such as PDA or ROP through GA-dependent mechanisms, whereas the effect on IVH may be mediated by GA-independent mechanisms.

Besides GA, several other factors potentially confound the association between CA and IVH. A number of studies provided data adjusted for confounding factors, but confounders accounted for in each model differed across studies. We performed separate analyses aggregating adjusted association measures. This reduced or made non-significant the association between CA and IVH (Supplementary Tables 3, 4). Earlier meta-analyses on the association between CA and cerebral palsy (Wu and Colford, 2000), BPD (Hartling et al., 2012), ROP (Villamor-Martinez et al., 2018a) also showed that the positive association found when unadjusted data were pooled, was reduced or became non-significant when only adjusted data were pooled. Moreover, in our previous meta-analysis on CA and PDA (Behbodi et al., 2016) we found that CA was risk factor for PDA when unadjusted data were pooled, and that CA was a protective factor for PDA when adjusted data were pooled.

Adjustment for potential confounders, particularly for GA and/or BW, is a common strategy used in observational studies analyzing predictors of outcomes of prematurity (Gagliardi et al., 2013). Quality assessment tools such as the NOS even downgrade studies for not adjusting for confounding factors. However, adjustment for GA and BW is controversial and can arguably lead to biased conclusions (Wilcox et al., 2011; Gagliardi et al., 2013). Preterm infants are at risk of adverse outcomes both due to their immaturity and due to the pathological conditions that led to their preterm birth (McElrath et al., 2008; Basso and Wilcox, 2010; Wilcox et al., 2011; Gagliardi et al., 2013). Very low GA is therefore both a risk factor for adverse outcomes, as well as a mediator in the causal pathway that links preterm birth to adverse outcomes (Wilcox et al., 2011; Gagliardi et al., 2013). The problem with adjusting for intermediate variables, such as GA, is that it may introduce bias unless each confounder is accounted for in the model (Hernández-Díaz et al., 2006; Basso and Wilcox, 2010; Wilcox et al., 2011; Gagliardi et al., 2013). As discussed by Gagliardi et al., “the difficulty of achieving—at least at the current level of knowledge of etiology of preterm birth—full control of all mediator–outcome confounders limits the possibility of causal interpretation of the associations found but not their descriptive value” (121, p. 798). In this sense, by providing analysis of both the unadjusted and adjusted data, our study may be a valuable contribution to the understanding of CA as etiopathogenic factor of both prematurity and IVH.

Our data suggest that CA-exposed infants are not only younger but also more clinically unstable than the non-exposed infants. This is reflected in the higher mortality and the higher rate of sepsis and PDA in CA-exposed infants (Table 1). Of note, meta-regression showed a correlation between the effect size of the association between CA and grade 3-4 IVH and the effect sizes of the association between CA and PDA. As mentioned elsewhere, the presence of a hemodynamically relevant PDA has been correlated with the occurrence of IVH and the proposed mechanism is the disturbance of cerebral blood flow (Ballabh, 2010, 2014; Inder et al., 2018; Poryo et al., 2018). Our data support this association between IVH and PDA in CA-exposed infants.

The biological plausibility of the association between CA and IVH is supported by the direct and indirect effects of inflammatory mediators. Hemodynamic disturbances in preterm infants with CA have been correlated with elevated cord blood concentrations of proinflammatory cytokines such as IL-6, IL-1beta and TNF-alpha (Yanowitz et al., 2002). Cytokines can act directly on the vascular smooth muscle, producing vascular relaxation and hypotension or indirectly by increasing the production of endothelium-derived vasoactive mediators (Yanowitz et al., 2002). In addition, cytokines can eventually promote a neuro-inflammatory cascade in the fetal brain penetrating across the blood brain barrier or activating specific receptors such as CD14 and TLR4 which are constitutively expressed in the circumventricular organs, choroid plexus and leptomeninges (Rivest, 2003; McAdams and Juul, 2012). Inflamed glial or endothelial cells, challenged by external stimuli, enhance the release/expression of various chemoattractants and adhesion molecules which may promote the platelet and neutrophil activation and adhesion determining possible endothelial cell damage and changes in blood rheology and flow (Stanimirovic and Satoh, 2000; Molina-Holgado and Molina-Holgado, 2010). These changes, occurring inside the fragile germinal matrix capillaries or within the vascular connection between germinal matrix and the subependymal venous network, may increase the likelihood of IVH in preterm infants with CA.

We have discussed the role of funisitis in earlier meta-analyses on CA and ROP (Villamor-Martinez et al., 2018a) and CA and PDA (Behbodi et al., 2016). It is worth noting that not all intraamniotic infections will induce an inflammatory response in the fetus (Revello et al., 2015). Funisitis is generally considered the histologic counterpart to fetal inflammatory response syndrome (Gantert et al., 2010; Revello et al., 2015). We found that exposure to funisitis did not significantly increase the risk of IVH, when compared to exposure to CA without funisitis. This is an argument against role of the fetal inflammatory response in the etiopathogenesis of IVH. We have previously reported that funisitis is not an additional risk factor for developing PDA (Behbodi et al., 2016) but the presence of funisitis significantly increased the risk of developing severe ROP (Villamor-Martinez et al., 2018a).

Our meta-analysis has several limitations that should be considered. Firstly, there was substantial heterogeneity in how CA was defined in studies. The definitions of clinical CA in particular varied substantially, and recent recommendations propose restricting the term CA to pathologic diagnosis (Higgins et al., 2016). Secondly, only 5 out of 85 included studies studied the association between CA and IVH as their main objective. However, this could also have reduced the effect of publication bias. Thirdly, only 13 out of the 85 included studies provided adjusted data, and they used different models and adjusted for different confounders. Finally, in this study and earlier studies on ROP (Villamor-Martinez et al., 2018a) and PDA (Behbodi et al., 2016), we had a much more limited number of studies to draw from for analyzing funisitis than when analyzing CA. The strengths of our study include: the use of a comprehensive search, duplication of screening, inclusion and data extraction to reduce bias, a large number of included studies, and an extensive analysis of confounding factors, through meta-analysis, meta-regression and the inclusion of adjusted data.

A significant limitation in any meta-analysis on IVH is the potential for heterogeneity in defining the condition. The grading system most commonly used for neonatal IVH was first reported by Papile et al. and later modified by Volpe and is based on the presence and amount of blood in the germinal matrix, lateral ventricles, and periventricular white matter (Volpe, 2015). Grade 1 represents germinal matrix hemorrhage only with no or minimal IVH (<10% of ventricular area on parasagittal view). When IVH occupies 10–50% of ventricular area on parasagittal view, it is defined as grade 2 (Volpe, 2015). Grade 3 is IVH with blood occupying more than 50% of the ventricular area on parasagittal view. Grade 4 represents severe IVH with associated periventricular echodensity (Volpe, 2015). Although grade 4 IVH is a periventricular hemorrhagic infarction rather than an extension of IVH *per se*, the 1–4 grading system remains pervasive in the literature and clinical setting despite debate regarding appropriate nomenclature (Leviton et al., 2007). In addition grade 3 and 4 IVHs are frequently grouped together as severe or high grade IVH (Leviton et al., 2007). Nevertheless, our meta-analysis shows a significant increased risk of both severe and less severe (grade 1–2) IVH in CA-exposed infants. Therefore, potential differences in IVH classification may not have affected the results.

## Conclusion

IVH is a multifactorial complication that is more common in more preterm and more clinically unstable infants. We established for the first time through meta-analysis that CA is a risk factor for IVH. We also confirmed earlier findings that CA is a risk factor for being born more preterm and presenting more clinical instability. However, in contrast to other complications of prematurity, such as PDA, ROP, or BPD (Hartling et al., 2012; Mitra et al., 2014; Behbodi et al., 2016; Villamor-Martinez et al., 2018a), the effect of CA on IVH appears to be independent of CA as a causative factor for very preterm birth.

## Data availability statement

The datasets generated and analyzed for this study can be found in the Harvard Dataverse (Harvard Dataverse, 2018): https://dataverse.harvard.edu/dataset.xhtml?persistentId=doi%3A10.7910%2FDVN%2FJ9RHUF.

A preprint version of this manuscript is made available to the scientific community on the preprint server bioRxiv (Villamor-Martinez et al., 2018b): https://www.biorxiv.org/content/early/2018/05/30/334375.

## Author contributions

EV-M carried out data collection, carried out statistical analyses, assessed methodological quality, contributed to interpretation of results, drafted the initial manuscript, and reviewed and revised the manuscript. MF contributed to the design of the study, the statistical analysis and interpretation of results and reviewed and revised the manuscript. OM selected studies for inclusion, carried out data collection and carried out statistical analyses. SP contributed to interpretation of results and reviewed and revised the manuscript. GC contributed to interpretation of results and reviewed and revised the manuscript. PD carried out and supervised data collection and contributed to interpretation of results. FM contributed to interpretation of results and reviewed and revised the manuscript. EV conceptualized and designed the study, carried out the search and selected studies for inclusion, supervised data collection, contributed to statistical analyses and interpretation of results, and reviewed and revised the manuscript. All authors approved the final manuscript as submitted.

### Conflict of interest statement

The authors declare that the research was conducted in the absence of any commercial or financial relationships that could be construed as a potential conflict of interest.
