# Corrigendum: Chorioamnionitis Is a Risk Factor for Intraventricular Hemorrhage in Preterm Infants: A Systematic Review and Meta-Analysis

**DOI:** 10.3389/fphys.2019.00102

**Published:** 2019-02-15

**Authors:** Eduardo Villamor-Martinez, Monica Fumagalli, Owais Mohammed Rahim, Sofia Passera, Giacomo Cavallaro, Pieter Degraeuwe, Fabio Mosca, Eduardo Villamor

**Affiliations:** ^1^Department of Pediatrics, School for Oncology and Developmental Biology (GROW), Maastricht University Medical Center, Maastricht, Netherlands; ^2^Neonatal Intensive Care Unit, Department of Clinical Sciences and Community Health, Fondazione IRCCS Cà Granda Ospedale Maggiore Policlinico, Università degli Studi di Milano, Milan, Italy

**Keywords:** chorioamnionitis, intraventricular hemorrhage, very preterm infant, systematic review, meta-analysis

In the original article, there was a mistake in Supplementary Table 1 and Supplementary Table 2 as published. The study by Vaihinger et al. was incorrectly given “8” points on the Newcastle Ottawa Scale instead of “9”. The corrected Supplementary Table 1 and Supplementary Table 2 appears below.

**Table 1:** Synoptic table of characteristics of all included studies.

**First author, year**	**Location (s)**	**Cohort/ case-control^a^**	**Perspective^a^**	**Prosp/Retro**	**Total infants (centers)**	**Mean BW (g)**	**Mean GA (wks)**	**Male (%)**	**ACS (%)**	**CA category^b^**	**Incidence of CA (%)**	**Definition of CA^c^**	**Incidence of all IVH (%)**	**Incidence of severe IVH (%)**	**Definition of IVH^d^**	**NO-S Quality score**
Aden, 2013	Sweden, Finland, USA	Ca-co	IVH	Prosp	612 (27)	844	26,2	59	100	CCA	23	NoDes		37	NA	6
Ahn, 2012	Seoul, Korea	Cohort	CA	Prosp	257 (1)	1536	30,6	60		HCA	35	Ref		2	Ref	8
Alexander, 1998	Dallas, USA	Cohort	CA	Prosp	1367 (1)	1138	28,9			CCA	7	Des		12	Ref	7
Arayici, 2004	Ankara, Turkey	Cohort	CA	Retro	281 (1)	1173	28,9	55	71	HCA	52	Des		10	Ref	7
Austeng, 2010	Sweden	Cohort	CA	Prosp	468 (7)	767	24,9	55	71	CCA	17	NoDes		14	Ref	8
Babnik, 2006	Ljubljana, Slovenia	Cohort	CA-IVH	Prosp	125 (1)	1019	27	54	53	HCA & F	39	Des	41		Ref	7
Barrera-Reyes, 2011	Mexico, Mexico	Cohort	CA	Prosp	104 (1)	1071	30,0	52		CCA	22	Ref	29		NA	6
Baumert, 2008	Katowice, Poland	Ca-co	IVH	Prosp	2675 (1)	3351	38,3	51		CCA	4	NoDes	15		Ref	7
Been, 2009	Rotterdam, Nehterlands	Cohort	CA	Prosp	301 (1)	1143	29,1	51	70	HCA	40	Des	15	4	NA	7
Bermick, 2016	Michigan, USA	Cohort	IVH	Retro	216	764	25,5	51	81	HCA	41	NoDes	56	NA	Ref	8
Bordigato, 2010	Padova, Italy	Cohort	CA	Prosp	29 (1)	805	26,7	59	76	HCA	48	Ref	27	3	NA	7
Botet, 2010	Spain	Ca-co	CA	Prosp	328 (12)	1057	28,2	54		HCA	50	Ref	27	12	NA	8
Bry, 2015	Gothenburg, Sweden	Cohort	CA	Prosp	24 (1)	777	25,9	50	100	HCA	67	Ref	17		NA	6
Dalton, 2015	Michigan, USA	Cohort	IVH	Retro	216 (1)	764	25,5	51	81	HCA	41	NoDes	56		Ref	6
Dempsey, 2005	Montreal, Canada	Cohort	CA	Retro	330 (1)	987	27,0		63	HCA	39	Des	10		Ref	7
Dexter, 2000	Rhode Island, USA	Cohort	CA	Prosp	275 (1)	904	26,5	53	22.3	HCA	57	Des	32	10	NA	6
Ecevit, 2014	Ankara, Turkey	Cohort	CA	Retro	36 (1)	1524	29,7	59		HCA	58	Des	19		NA	6
Elimian, 2000	New York, USA	Cohort	CA	Prosp	1260 (1)	1183	29.0		42	HCA	42	Ref	27	12	Ref	9
Erdemir, 2013	Izkir, Turkey	Cohort	CA	Prosp	57 (1)	1675	30,8	46	65	HCA and/or CCA	21	Des	12		NA	6
Fung, 2003	Clayton, Australia	Cohort	CA	Prosp	62 (1)	794	26,2	50	83	HCA and/or CCA	25	Des		24	NA	6
Gagliardi, 2014	Italian Neonatal Network	Cohort	CA	Retro	3606 (82)	938	27,4	50	84	CCA	42	NoDes		11	Ref	9
Garcia-Munoz Rodrigo, 2014	Spanish Network	Cohort	CA	Prosp	8330 (53)	1086	28,5	52	87	CCA	18	Des		10	NA	9
Gawade, 2013	Springfield, USA	Cohort	IVH	Retro	78 (1)	980	26,8	59	85	CCA	15	Nodes	44	12	Ref	6
Gonzalez-Luis, 2002	Barcelona, Spain	Ca-co	CA	Retro	135 (1)	1147	28,9			CCA	33	Des	20	7	NA	7
Gray, 1997	Brisbane, Australia	Cohort	IVH	Retro	158 (1)	955	27,0	57		HCA and/or CCA	10	Des	25	8	NA	7
Hendson, 2011	Edmonton, Canada	Cohort	CA	Prosp	617 (1)	930	26,9	48	83	HCA	48	Des		16	Ref	7
Hitti, 2001	Seattle, USA	Cohort	CA	Prosp	140 (2)	1699	29,3		51	Microbiol	17	Des	14	6	Ref	7
Holcroft, 2003	Baltimore, USA	Ca-Co	CA	Retro	213 (1)	1045	28,3			CCA	21	Des	36		NA	5
Kallankari, 2010	Oulu, Finland	Cohort	IVH	Prosp	163 (1)		92,2		86	HCA	39	Ref	14		Ref	7
Kaulkola,2006	Oulu, Finland	Cohort	IVH	Prosp	51 (1)	772	27	41	90	HCAA	49	Ref	22		Ref	7
Kidokoro	New Zealand, Australia, USA	Cohort	IVH	Prosp	325 (3)	959	27,5	47	86	CCA	22	Des	19	4	Ref	8
Kim, 2015	Seoul, Korea	Cohort	CA	Retro	235 (1)	1104	29,2	50	81	HCA & F	38	Ref		6	NA	7
Kirchner, 2007	Vienna, Austria	Cohort	CA	Retro	44 (1)		27,9	53	93	Microbiol	34	NoDes		9	Ref	7
Klebermans-Schrehof	Vienna, Austria	Cohort	IVH	Retro	471 (1)	996	27,4	53	93	HCA and/or CCA	38	NoDes	32		Ref	7
Kosuge, 2000	Minamikawac hi-machi, Japan	Cohort	CA	Retro	81 (1)	1181	28,1	68	17	HCA	54	Ref	11		NA	6
Lau, 2005	Vancouver, Canada	Cohort	CA	Prosp	1296 (1)	2068	33,2	55	47	HCA & F	31	Ref		5	Ref	7
Lee Hyun Ju, 2011	Seoul, Korea	Cohort	CA	Retro	147 (2)	791	27	55	67	HCA	48	Ref	40		Ref	7
Lee Ju Young, 2010	Seoul, Korea	Ca-Co	IVH	Retro	177 (2)	954	27,5	53		HCA	45	NoDes		22	Ref	8
Lim, 2011	Taiwan	Ca-Co	IVH	Retro	72 (1)	768	24,7	64		CCA	13	Des		50	Ref	9
Linder, 2003	Israel	Ca-Co	IVH	Retro	105 (1)	826	25,4	58	73	CCA	24	Des		34	Ref	9
Liu, 2014	Changhai, China	Cohort	CA	Prosp	95 (1)	1706	31,7	58	89	HCA	52	Ref	43	8	Ref	7
Logan, 2013	USA	Cohort	IVH	Retro	921 (14)			51	90	HCA	36	Des	6		NA	7
Lu, 2016	Jiangsu, China	Ca-Co	IVH	Retro	137 (1)	1205	31,9	58	50	HCA & CCA	44	Des	24		Ref	7
Mehta, 2006	New Brunswick, USA	Cohort	CA-IVH	Retro	164 (1)					HCA	39	Ref	37		NA	7
Mestan, 2010	Boston, USA	Cohort	CA	Prosp	256 (1)	1437	30,3	48	77	HCA	37	Ref		4	Ref	7
Miyazaki, 2016	Network database, Japan	Cohort	CA	Retro	4078 (54)	973	27,6	49	41	HCA	30	Ref	15		Ref	9
Morales, 1987	Orlando, USA	Ca-Co	Ca	Prosp	86 (1)	1178	29,2			HCA & CCA	50	Des	86	28	Ref	7
Mu, 2008	Taipei, Taiwan	Cohort	CA	Prosp	119 (1)	1108	28,6	54	45	HCA	54	Ref	22	17	Ref	8
Nasef, 2013	Toronto, Canada	Cohort	CA	Retro	274 (1)	952	27	55	85	HCA & CCA	47	Ref	23	1	Ref	7
Ogunyemi, 2003	New jersey, USA	Cohort	CA	Retro	774 (1)	1313	29,4		53	HCA	33	Ref	39	5	NA	7
Oh, 2015	Seoul, Korea	Cohort	CA	Retro	175 (1)	765	27,1	55	60	HCA	25	Ref		NA	Ref	9
Oh, 2018	Seoul, Korea	Cohort	IVH	Retro	207	1269	29.7	48	75	HCA	44	Ref	7*	NA	Ref	9
Ohyama, 2002	Yokohama, Japan	Cohort	CA	Retro	143 (1)	1162	27,8			HCA & F	63	Ref	9		NA	6
Osmanagaoglu, 2005	Trabzon, Turkey	Cohort	CA	Retro	254 (1)	1828	32	56	65	CCA	12	Ref		6	NA	7
Pappas, 2014	USA	Cohort	CA	Prosp	1918 (16)		24,4	51	75	HCA	55	Ref		29	NA	6
Perrone, 2012	Siena, Italy	Cohort	CA	Prosp	92 (1)	998	26,3			HCA	49	Ref	48		Ref	7
Polam, 2005	New Brunswick, USA	Cohort	CA	Prosp	177 (1)	955	26,5	53	74	HCA	58	Des	26	7	NA	6
Poralla, 2012	Bonn, Germany	Cohort	IVH	Retro	132 (1)	714	25,5	50	87	CCA	39	Des	44		NA	6
Richardson, 2006	London Ontario, Canada	Cohort	CA	Retro	660 (1)	1602	30,1	55		HCA	44	Des	22		NA	6
Rocha, 2006 & Rocha, 2007	Porto, Portugal	Cohort	CA	Retro	452 (3)	1504	29,5	52	65	HCA	28	Ref	18	9	Ref	9
Rodríguez-Trujillo, 2016	Barcelona, Spain	Cohort	CA	Prosp	165 (1)	1721	30,2			HCA	67	NoDes		10	NA	7
Rong, 2012	Wuhan, China	Ca-co	IVH	Retro	232 (3)	1566	30,7	73	41	CCA	19	NoDes	34		NA	8
Ryckman, 2011	Iowa, USA	Cohort	IVH	Prosp	219			58		CCA	15	NoDes	22		Ref	6
Salas, 2013	Alabama, USA	Cohort	CA	Retro	347 (1)	829	26,1	50	62	HCA	43	Ref		17	Ref	7
Sarkar, 2005	New York, USA	Cohort	CA-IVH	Prosp	62 (1)	884	62,2	45	90	HCA	47	Ref	15	7	Ref	7
Sato, 2011	Yokohama, Japan	Cohort	CA	Retro	302 (1)	938,4	26,3	52	62	HCA	52	Ref	27		NA	7
Seliga-Siwecka, 2013	Warsaw, Poland	Cohort	CA	Prosp	383 (1)	1338	29,2	56	84	HCA	37	Ref		45	Ref	9
Shankaran, 2014	USA and Sweden	Ca-co	IVH	Prosp	1111 (24)	817	26,0	56	53	CCA	29	NoDes	52		Ref	8
Smit, 2015	Veldhoven, Netherlands	Cohort	CA	Retro	300 (1)	1303	29,4	54	92	HCA & F	45	Ref		4	NA	7
Soraisham, 2009	Canadian Neonatal Network	Cohort	CA	Prosp	3094 (24)	1320	28,9	53	79	CCA	15	Des		14	Ref	9
Soraisham, 2013	Regional NICU Southern Alberta, Canada	Cohort	CA	Retro	384 (1)	885	26,3	51	86	HCA	51	Des	21	7	Ref	6
Suarez, 2001	Chicago, USA	Ca-co	IVH	Retro	280 (1)	1328	29,6	56		CCA	19	Des	20		Ref	7
Suppiej, 2009	Padova, Italy	Cohort	CA	Prosp	104 (1)	1078	28,5	46	87	HCA	39	Ref	13		Ref	6
Trevisanuto, 2010	Padua, Italy	Ca-co	CA	Prosp	142 (1)	1075	27,8	55	89	HCA	50	Ref		4	Ref	8
Tsiartas, 2013	Králove, Czech Republic	Cohort	CA	Retro	231 (1)	1975	33,0		56	HCA & F	61	Ref	20	1	Ref	7
Vaihinger, 2013	Buenos Aires, Argentina	Ca-co	IVH	Retro	198 (1)	1072	28,0	53	66	CCA	24	NoDes	25		Ref	9
van Vliet, 2012	Amsterdam, Netherlands	Cohort	CA	Prosp	72 (1)	1110	29,0	51	82	HCA	29	Ref	29		NA	6
Vergani, 2004	Monza, Italy	Cohort	IVH	Retro	653 (1)	1335	30,1	50	50	CCA	11	Des	7		Ref	7
Watterberg, 1999	Pennsylvania, USA	Cohort	CA	Prosp	40 (2)	751	25,3	38	85	HCA	55	NoDes	38	8	Na	7
Wirbelauer, 2011	Wuerzburg, Germany	Cohort	CA	Prosp	71 (1)	871	27,9	52	94	HCA & F	24	Ref		9	Ref	7
Xu, 2012	Hangzhou, China	Cohort	IVH	Prosp	88 (1)	1540	31,8	53		HCA and/or CCA	47	NoDes	25	8	Ref	7
Yamada, 2015	Miyazaki, Japan	Cohort	CA	Prosp	212 (1)		25			HCA	65	Ref		20	NA	7
Yanowitz, 2006	Pittsburgh, USA	Cohort	CA	Prosp	49 (1)	1273	28,7	61		HCA	49	Ref	57	2	Ref	7
Yoon, 1995	Seoul, Korea	Cohort	CA	Prosp	50 (1)	1852	31,8			HCA	58	Ref	30		NA	7
Zanardo, 2008	Padua, Italy	Cohort	CA-IVH	Prosp	287 (1)	1146	29,3	48	80	HCA	24	Ref	12	1	Ref	7

*CA, chorioamnionitis; IVH, intraventricular hemorrhage; NOS, Newcastle-Ottawa Scale; ACS, antenatal corticosteroids*.

a Abbreviations for study design: Ca-Co, case-control study; Perspective, CA, study analyzed IVH as outcome of chorioamnionitis; Perspective, IVH, study analyzed chorioamnionitis as risk factor for IVH; Perspective, CA-IVH, study analyzed the association between chorioamnionitis and IVH as primary outcome. Prosp, prospective; Retro, retrospective;

b *Chorioamnionitis category: CCA, clinical chorioamnionitis; HCA, histological chorioamnionitis; HCA & F, histological chorioamnionitis with funisitis mentioned separately. HCA and/or CCA, chorioamnionitis defined as positive when infants had histological or clinical chorioamnionitis; Microbiol, microbiological chorioamnionitis*.

c *Definition of chorioamnionitis: NoDes, no description; Des, clinical or histological description; Ref, defined according to cited article*.

d *Definition of ROP: ICROP, International Classification of Retinopathy of Prematurity; Ref, defined according to cited article; Treat, laser treatment of ROP; NA, no diagnostic criteria mentioned*.

**Table 2:** Newcastle-Ottawa Quality assessment of included studies.

**First author, year**	**Perspective**	**Select**.	**Comp**.	**Outc**.	**Total**	**Reason for downgrade**
Aden, 2013	IVH	4	0	2	6	No adjustment
Ahn, 2013	CA	4	1	3	8	Only adjusted for 1 factor
Alexander, 1998	CA	4	0	3	7	No adjustment
Arayici, 2004	CA	4	0	3	7	No adjustment
Austeng, 2010	CA	3	2	3	8	No CA definition
Babnik, 2006	CA-IVH	4	0	3	7	No adjustment
Barrera-Reyes, 2011	CA	4	0	2	6	No adjustment
Baumert, 2008	IVH	4	0	3	7	No adjustment
Been, 2009	CA	4	0	3	7	No adjustment
Bermick, 2016	IVH	3	2	3	8	No CA definition
Bordigato, 2010	CA	4	0	3	7	No adjustment
Botet, 2010	CA	4	1	3	8	Only adjusted for 1 factor
Bry, 2015	CA	4	0	2	6	No adjustment
Dalton, 2015	IVH	3	0	3	6	No CA definition
Dempsey, 2005	CA	4	0	3	7	No adjustment
Dexter, 2000	CA	4	0	2	6	Loss to follow up, no adjustment
Ecevit, 2014	CA	4	0	2	6	No IVH definition, no adjustment
Elimian, 2000	CA	4	2	3	9	
Erdemir, 2013	CA	4	0	2	6	No IVH definition, no adjustment
Fung, 2003	CA	4	0	2	6	No IVH definition, no adjustment
Gagliardi, 2014	CA	4	2	3	9	
Garcia-Munoz Rodrigo, 2014	CA	4	2	3	9	
Gawade, 2013	IVH	3	0	3	6	No CA definition, no adjustment
Gonzalez-Luis, 2002	CA	4	0	3	7	No adjustment
Gray, 1997	CA	4	0	3	7	
Hendson, 2011	CA	4	0	3	7	No adjustment
Hitti, 2001	CA	4	0	3	7	No adjustment
Holcroft, 2003	IVH	2	0	3	5	No IVH definition, no adjustment
Kallankari, 2010	IVH	4	0	3	7	No adjustment
Kaulkola,2006	IVH	4	0	3	7	No adjustment
Kidokoro	IVH	4	1	3	8	Only adjusted for 1 factor
Kim, 2015	CA	4	0	3	7	No adjustment
Kirchner, 2007	CA	4	0	3	7	No adjustment
Klebermans-Schrehof	IVH	4	0	3	7	No adjustment
Kosuge, 2000	CA	4	0	2	6	No IVH definition, no adjustment
Lau, 2005	CA	4	0	3	7	No adjustment
Lee Hyun Ju, 2011	CA	4	0	3	7	No adjustment
Lee Ju Young, 2010	IVH	4	1	3	8	Only adjusted for 1 factor
Lim, 2011	IVH	4	2	3	9	
Linder, 2003	IVH	4	2	3	9	
Liu, 2014	CA	4	0	3	7	No adjustment
Logan, 2013	IVH	4	0	3	7	No adjustment
Lu, 2016	IVH	4	0	3	7	No adjustment
Mehta, 2006	CA-IVH	4	0	3	7	No adjustment
Mestan, 2010	CA	4	0	3	7	No adjustment
Miyazaki, 2016	CA	4	2	3	9	
Morales, 1987	CA	4	0	3	7	No adjustment
Mu, 2008	CA	4	1	3	8	Only adjusted for 1 factor
Nasef, 2013	CA	4	0	3	7	No adjustment
Ogunyemi, 2003	CA	4	0	3	7	No adjustment
Oh, 2015	IVH	4	2	3	9	
Oh, 2018	CA	4	2	3	9	
Ohyama, 2002	CA	4	0	2	6	No IVH definition, no adjustment
Osmanagaoglu, 2005	CA	4	0	3	7	No adjustment
Pappas, 2014	CA	4	0	2	6	No IVH definition, no adjustment
Perrone, 2012	CA	4	0	3	7	No IVH definition
Polam, 2005	CA	4	0	2	6	Loss to follow up, no adjustment
Poralla, 2012	IVH	4	0	2	6	No IVH definition
Richardson, 2006	CA	4	0	2	6	No IVH definition, no adjustment
Rocha, 2006 & Rocha, 2007	CA	4	2	3	9	
Rodríguez-Trujillo, 2016	CA	4	0	3	7	No adjustment
Rong, 2012	IVH	4	2	2	8	No CA definition
Ryckman, 2011	IVH	3	0	3	6	No CA definition, no adjustment
Salas, 2013	CA	4	0	3	7	No adjustment
Sarkar, 2005	CA-IVH	4	0	3	7	No adjustment
Sato, 2011	CA	4	1	2	7	No IVH definition, only adjusted for 1 factor
Seliga-Siwecka, 2013	CA	4	2	3	9	
Shankaran, 2014	IVH	4	2	2	8	
Smit, 2015	CA	4	0	3	7	No adjustment
Soraisham, 2009	CA	4	2	3	9	
Soraisham, 2013	CA	4	0	2	6	Loss to follow up, no adjustment
Suarez, 2001	IVH	4	0	3	7	No adjustment
Suppiej, 2009	CA	4	0	2	6	Loss to follow up, no adjustment
Trevisanuto, 2010	CA	4	1	3	8	Only adjusted for 1 factor
Tsiartas, 2013	CA	4	0	3	7	No adjustment
Vaihinger, 2013	IVH	4	2	3	9	
van Vliet, 2012	CA	4	0	2	6	No adjustment
Vergani, 2004	IVH	4	0	3	7	No adjustment
Watterberg, 1999	CA	4	0	3	7	No adjustment
Wirbelauer, 2011	CA	4	0	3	7	No adjustment
Xu, 2012	CA	4	0	3	7	No adjustment
Yamada, 2015	CA	4	0	3	7	No adjustment
Yanowitz, 2006	CA	4	0	3	7	No adjustment
Yoon, 1995	CA	4	0	3	7	No adjustment
Zanardo, 2008	CA-IVH	4	0	3	7	No adjustment

*Select., selection; Comp., comparability; Outc., outcome; CA, chorioamnionitis; IVH, intraventricular hemorrhage*.

The authors apologize for this error and state that this does not change the scientific conclusions of the article in any way. The original article has been updated.

