# Supplementary material for: Chorioamnionitis Is a Risk Factor for Intraventricular Hemorrhage in Preterm Infants: A Systematic Review and Meta-Analysis
Source: Front Physiol. 2018 Sep 11;9:1253. doi: 10.3389/fphys.2018.01253 (PMC6142185; doi:10.3389/fphys.2018.01253)
Supplement: Supplementary file 1 [file Data_Sheet_1.pdf]

Supplementary Material

Chorioamnionitis Is a Risk Factor for Intraventricular Hemorrhage in Preterm Infants: A Systematic Review and Meta-Analysis

Eduardo Villamor-Martinez[1], Monica Fumagalli[2], Owais Mohammed Rahim[1], Sofia Passera[2], Giacomo Cavallaro[2], Pieter Degraeuwe[1], Fabio Mosca,[2] Eduardo Villamor[1*]

[1]Department of Pediatrics, Maastricht University Medical Center (MUMC+), School for Oncology and Developmental Biology (GROW), Maastricht, the Netherlands.

[2]Neonatal Intensive Care Unit, Department of Clinical Sciences and Community Health, Fondazione IRCCS Cà Granda Ospedale Maggiore Policlinico, Università degli Studi di Milano, 20122 Milan, Italy

***Correspondence**
Eduardo Villamor
e.villamor@mumc.nl

1 Supplementary Data

1.1 Supplementary Tables

Supplementary Table 1. Synoptic table of characteristics of all included studies

First author, year	Location(s)	Cohort/ case-control[a]	Perspective[a]	Prosp/ Retro	Total infants (centers)	Mean BW (g)	Mean GA (wks)	Male (%)	ACS (%)	CA category[b]	Incidence of CA (%)	Definition of CA[c]	Incidence of all IVH (%)	Incidence of severe IVH (%)	Definition of IVH[d]	NOS Quality score
Aden, 2013	Sweden, Finland, USA	Ca-co	IVH	Prosp	612 (27)	844	26,2	59	100	CCA	23	NoDes		37	NA	6
Ahn, 2012	Seoul, Korea	Cohort	CA	Prosp	257 (1)	1536	30,6	60		HCA	35	Ref		2	Ref	8
Alexander, 1998	Dallas, USA	Cohort	CA	Prosp	1367 (1)	1138	28,9			CCA	7	Des		12	Ref	7
Arayici, 2004	Ankara, Turkey	Cohort	CA	Retro	281 (1)	1173	28,9	55	71	HCA	52	Des		10	Ref	7
Austeng, 2010	Sweden	Cohort	CA	Prosp	468 (7)	767	24,9	55	71	CCA	17	NoDes		14	Ref	8
Babnik, 2006	Ljubljana, Slovenia	Cohort	CA-IVH	Prosp	125 (1)	1019	27	54	53	HCA & F	39	Des	41		Ref	7
Barrera-Reyes, 2011	Mexico, Mexico	Cohort	CA	Prosp	104 (1)	1071	30,0	52		CCA	22	Ref	29		NA	6
Baumert, 2008	Katowice, Poland	Ca-co	IVH	Prosp	2675 (1)	3351	38,3	51		CCA	4	NoDes	15		Ref	7
Been, 2009	Rotterdam, Nehterlands	Cohort	CA	Prosp	301 (1)	1143	29,1	51	70	HCA	40	Des	15	4	NA	7
Bermick, 2016	Michigan, USA	Cohort	IVH	Retro	216	764	25,5	51	81	HCA	41	NoDes	56	NA	Ref	8
Bordigato, 2010	Padova, Italy	Cohort	CA	Prosp	29 (1)	805	26,7	59	76	HCA	48	Ref	27	3	NA	7
Botet, 2010	Spain	Ca-co	CA	Prosp	328 (12)	1057	28,2	54		HCA	50	Ref	27	12	NA	8
Bry, 2015	Gothenburg, Sweden	Cohort	CA	Prosp	24 (1)	777	25,9	50	100	HCA	67	Ref	17		NA	6
Dalton, 2015	Michigan, USA	Cohort	IVH	Retro	216 (1)	764	25,5	51	81	HCA	41	NoDes	56		Ref	6

First author, year	Location(s)	Cohort/ case-control[a]	Perspective[a]	Prosp/ Retro	Total infants (centers)	Mean BW (g)	Mean GA (wks)	Male (%)	ACS (%)	CA category[b]	Incidence of CA (%)	Definition of CA[c]	Incidence of all IVH (%)	Incidence of severe IVH (%)	Definition of IVH[d]	NOS Quality score
Dempsey, 2005	Montreal, Canada	Cohort	CA	Retro	330 (1)	987	27,0		63	HCA	39	Des	10		Ref	7
Dexter, 2000	Rhode Island, USA	Cohort	CA	Prosp	275 (1)	904	26,5	53	22.3	HCA	57	Des	32	10	NA	6
Ecevit, 2014	Ankara, Turkey	Cohort	CA	Retro	36 (1)	1524	29,7	59		HCA	58	Des	19		NA	6
Elimian, 2000	New York, USA	Cohort	CA	Prosp	1260 (1)	1183	29.0		42	HCA	42	Ref	27	12	Ref	9
Erdemir, 2013	Izkir, Turkey	Cohort	CA	Prosp	57 (1)	1675	30,8	46	65	HCA and/or CCA	21	Des	12		NA	6
Fung, 2003	Clayton, Australia	Cohort	CA	Prosp	62 (1)	794	26,2	50	83	HCA and/or CCA	25	Des		24	NA	6
Gagliardi, 2014	Italian Neonatal Network	Cohort	CA	Retro	3606 (82)	938	27,4	50	84	CCA	42	NoDes		11	Ref	9
Garcia-Munoz Rodrigo, 2014	Spanish Network	Cohort	CA	Prosp	8330 (53)	1086	28,5	52	87	CCA	18	Des		10	NA	9
Gawade, 2013	Springfield, USA	Cohort	IVH	Retro	78 (1)	980	26,8	59	85	CCA	15	Nodes	44	12	Ref	6
Gonzalez-Luis, 2002	Barcelona, Spain	Ca-co	CA	Retro	135 (1)	1147	28,9			CCA	33	Des	20	7	NA	7
Gray, 1997	Brisbane, Australia	Cohort	IVH	Retro	158 (1)	955	27,0	57		HCA and/or CCA	10	Des	25	8	NA	7
Hendson, 2011	Edmonton, Canada	Cohort	CA	Prosp	617 (1)	930	26,9	48	83	HCA	48	Des		16	Ref	7
Hitti, 2001	Seattle, USA	Cohort	CA	Prosp	140 (2)	1699	29,3		51	Microbiol	17	Des	14	6	Ref	7
Holcroft, 2003	Baltimore, USA	Ca-Co	CA	Retro	213 (1)	1045	28,3			CCA	21	Des	36		NA	5
Kallankari, 2010	Oulu, Finland	Cohort	IVH	Prosp	163 (1)		92,2		86	HCA	39	Ref	14		Ref	7
Kaulkola,2006	Oulu, Finland	Cohort	IVH	Prosp	51 (1)	772	27	41	90	HCAA	49	Ref	22		Ref	7

First author, year	Location(s)	Cohort/ case-control[a]	Perspective[a]	Prosp/ Retro	Total infants (centers)	Mean BW (g)	Mean GA (wks)	Male (%)	ACS (%)	CA category[b]	Incidence of CA (%)	Definition of CA[c]	Incidence of all IVH (%)	Incidence of severe IVH (%)	Definition of IVH[d]	NOS Quality score
Kidokoro	New Zealand, Australia, USA	Cohort	IVH	Prosp	325 (3)	959	27,5	47	86	CCA	22	Des	19	4	Ref	8
Kim, 2015	Seoul, Korea	Cohort	CA	Retro	235 (1)	1104	29,2	50	81	HCA & F	38	Ref		6	NA	7
Kirchner, 2007	Vienna, Austria	Cohort	CA	Retro	44 (1)		27,9	53	93	Microbiol	34	NoDes		9	Ref	7
Klebermans-Schrehof	Vienna, Austria	Cohort	IVH	Retro	471 (1)	996	27,4	53	93	HCA and/or CCA	38	NoDes	32		Ref	7
Kosuge, 2000	Minamikawachi-machi, Japan	Cohort	CA	Retro	81 (1)	1181	28,1	68	17	HCA	54	Ref	11		NA	6
Lau, 2005	Vancouver, Canada	Cohort	CA	Prosp	1296 (1)	2068	33,2	55	47	HCA & F	31	Ref		5	Ref	7
Lee Hyun Ju, 2011	Seoul, Korea	Cohort	CA	Retro	147 (2)	791	27	55	67	HCA	48	Ref	40		Ref	7
Lee Ju Young, 2010	Seoul, Korea	Ca-Co	IVH	Retro	177 (2)	954	27,5	53		HCA	45	NoDes		22	Ref	8
Lim, 2011	Taiwan	Ca-Co	IVH	Retro	72 (1)	768	24,7	64		CCA	13	Des		50	Ref	9
Linder, 2003	Israel	Ca-Co	IVH	Retro	105 (1)	826	25,4	58	73	CCA	24	Des		34	Ref	9
Liu, 2014	Changhai, China	Cohort	CA	Prosp	95 (1)	1706	31,7	58	89	HCA	52	Ref	43	8	Ref	7
Logan, 2013	USA	Cohort	IVH	Retro	921 (14)			51	90	HCA	36	Des	6		NA	7
Lu, 2016	Jiangsu, China	Ca-Co	IVH	Retro	137 (1)	1205	31,9	58	50	HCA & CCA	44	Des	24		Ref	7
Mehta, 2006	New Brunswick, USA	Cohort	CA-IVH	Retro	164 (1)					HCA	39	Ref	37		NA	7
Mestan, 2010	Boston, USA	Cohort	CA	Prosp	256 (1)	1437	30,3	48	77	HCA	37	Ref		4	Ref	7
Miyazaki, 2016	Network database, Japan	Cohort	CA	Retro	4078 (54)	973	27,6	49	41	HCA	30	Ref	15		Ref	9
Morales, 1987	Orlando, USA	Ca-Co	Ca	Prosp	86 (1)	1178	29,2			HCA & CCA	50	Des	86	28	Ref	7
Mu, 2008	Taipei, Taiwan	Cohort	CA	Prosp	119 (1)	1108	28,6	54	45	HCA	54	Ref	22	17	Ref	8

First author, year	Location(s)	Cohort/ case-control[a]	Perspective[a]	Prosp/ Retro	Total infants (centers)	Mean BW (g)	Mean GA (wks)	Male (%)	ACS (%)	CA category[b]	Incidence of CA (%)	Definition of CA[c]	Incidence of all IVH (%)	Incidence of severe IVH (%)	Definition of IVH[d]	NOS Quality score
Nasef, 2013	Toronto, Canada	Cohort	CA	Retro	274 (1)	952	27	55	85	HCA & CCA	47	Ref	23	1	Ref	7
Ogunyemi, 2003	New jersey, USA	Cohort	CA	Retro	774 (1)	1313	29,4		53	HCA	33	Ref	39	5	NA	7
Oh, 2015	Seoul, Korea	Cohort	CA	Retro	175 (1)	765	27,1	55	60	HCA	25	Ref		NA	Ref	9
Oh, 2018	Seoul, Korea	Cohort	IVH	Retro	207	1269	29.7	48	75	HCA	44	Ref	7*	NA	Ref	9
Ohyama, 2002	Yokohama, Japan	Cohort	CA	Retro	143 (1)	1162	27,8			HCA & F	63	Ref	9		NA	6
Osmanagaoglu, 2005	Trabzon, Turkey	Cohort	CA	Retro	254 (1)	1828	32	56	65	CCA	12	Ref		6	NA	7
Pappas, 2014	USA	Cohort	CA	Prosp	1918 (16)		24,4	51	75	HCA	55	Ref		29	NA	6
Perrone, 2012	Siena, Italy	Cohort	CA	Prosp	92 (1)	998	26,3			HCA	49	Ref	48		Ref	7
Polam, 2005	New Brunswick, USA	Cohort	CA	Prosp	177 (1)	955	26,5	53	74	HCA	58	Des	26	7	NA	6
Poralla, 2012	Bonn, Germany	Cohort	IVH	Retro	132 (1)	714	25,5	50	87	CCA	39	Des	44		NA	6
Richardson, 2006	London Ontario, Canada	Cohort	CA	Retro	660 (1)	1602	30,1	55		HCA	44	Des	22		NA	6
Rocha, 2006 & Rocha, 2007	Porto, Portugal	Cohort	CA	Retro	452 (3)	1504	29,5	52	65	HCA	28	Ref	18	9	Ref	9
Rodríguez-Trujillo, 2016	Barcelona, Spain	Cohort	CA	Prosp	165 (1)	1721	30,2			HCA	67	NoDes		10	NA	7
Rong, 2012	Wuhan, China	Ca-co	IVH	Retro	232 (3)	1566	30,7	73	41	CCA	19	NoDes	34		NA	8
Ryckman, 2011	Iowa, USA	Cohort	IVH	Prosp	219			58		CCA	15	NoDes	22		Ref	6
Salas, 2013	Alabama, USA	Cohort	CA	Retro	347 (1)	829	26,1	50	62	HCA	43	Ref		17	Ref	7
Sarkar, 2005	New York, USA	Cohort	CA-IVH	Prosp	62 (1)	884	62,2	45	90	HCA	47	Ref	15	7	Ref	7
Sato, 2011	Yokohama, Japan	Cohort	CA	Retro	302 (1)	938,4	26,3	52	62	HCA	52	Ref	27		NA	7

First author, year	Location(s)	Cohort/ case-control[a]	Perspective[a]	Prosp/ Retro	Total infants (centers)	Mean BW (g)	Mean GA (wks)	Male (%)	ACS (%)	CA category[b]	Incidence of CA (%)	Definition of CA[c]	Incidence of all IVH (%)	Incidence of severe IVH (%)	Definition of IVH[d]	NOS Quality score
Seliga-Siwecka, 2013	Warsaw, Poland	Cohort	CA	Prosp	383 (1)	1338	29,2	56	84	HCA	37	Ref		45	Ref	9
Shankaran, 2014	USA and Sweden	Ca-co	IVH	Prosp	1111 (24)	817	26,0	56	53	CCA	29	NoDes	52		Ref	8
Smit, 2015	Veldhoven, Netherlands	Cohort	CA	Retro	300 (1)	1303	29,4	54	92	HCA & F	45	Ref		4	NA	7
Soraisham, 2009	Canadian Neonatal Network	Cohort	CA	Prosp	3094 (24)	1320	28,9	53	79	CCA	15	Des		14	Ref	9
Soraisham, 2013	Regional NICU Southern Alberta, Canada	Cohort	CA	Retro	384 (1)	885	26,3	51	86	HCA	51	Des	21	7	Ref	6
Suarez, 2001	Chicago, USA	Ca-co	IVH	Retro	280 (1)	1328	29,6	56		CCA	19	Des	20		Ref	7
Suppiej, 2009	Padova, Italy	Cohort	CA	Prosp	104 (1)	1078	28,5	46	87	HCA	39	Ref	13		Ref	6
Trevisanuto, 2010	Padua, Italy	Ca-co	CA	Prosp	142 (1)	1075	27,8	55	89	HCA	50	Ref		4	Ref	8
Tsiartas, 2013	Králove, Czech Republic	Cohort	CA	Retro	231 (1)	1975	33,0		56	HCA & F	61	Ref	20	1	Ref	7
Vaihinger, 2013	Buenos Aires, Argentina	Ca-co	IVH	Retro	198 (1)	1072	28,0	53	66	CCA	24	NoDes	25		Ref	9
van Vliet, 2012	Amsterdam, Netherlands	Cohort	CA	Prosp	72 (1)	1110	29,0	51	82	HCA	29	Ref	29		NA	6
Vergani, 2004	Monza, Italy	Cohort	IVH	Retro	653 (1)	1335	30,1	50	50	CCA	11	Des	7		Ref	7
Watterberg, 1999	Pennsylvania, USA	Cohort	CA	Prosp	40 (2)	751	25,3	38	85	HCA	55	NoDes	38	8	Na	7
Wirbelauer, 2011	Wuerzburg, Germany	Cohort	CA	Prosp	71 (1)	871	27,9	52	94	HCA & F	24	Ref		9	Ref	7
Xu, 2012	Hangzhou, China	Cohort	IVH	Prosp	88 (1)	1540	31,8	53		HCA and/or CCA	47	NoDes	25	8	Ref	7
Yamada, 2015	Miyazaki, Japan	Cohort	CA	Prosp	212 (1)		25			HCA	65	Ref		20	NA	7

First author, year	Location(s)	Cohort/ case-control[a]	Perspective[a]	Prosp/ Retro	Total infants (centers)	Mean BW (g)	Mean GA (wks)	Male (%)	ACS (%)	CA category[b]	Incidence of CA (%)	Definition of CA[c]	Incidence of all IVH (%)	Incidence of severe IVH (%)	Definition of IVH[d]	NOS Quality score
Yanowitz, 2006	Pittsburgh, USA	Cohort	CA	Prosp	49 (1)	1273	28,7	61		HCA	49	Ref	57	2	Ref	7
Yoon, 1995	Seoul, Korea	Cohort	CA	Prosp	50 (1)	1852	31,8			HCA	58	Ref	30		NA	7
Zanardo, 2008	Padua, Italy	Cohort	CA-IVH	Prosp	287 (1)	1146	29,3	48	80	HCA	24	Ref	12	1	Ref	7

CA: chorioamnionitis; IVH: intraventricular hemorrhage; NOS: Newcastle-Ottawa Scale; ACS: antenatal corticosteroids.

[a]Abbreviations for study design: Ca-Co: case-control study; Perspective, CA: study analyzed IVH as outcome of chorioamnionitis; Perspective, IVH: study analyzed chorioamnionitis as risk factor for IVH; Perspective, CA-IVH: study analyzed the association between chorioamnionitis and IVH as primary outcome. Prosp: prospective; Retro: retrospective;

[b]Chorioamnionitis category: CCA: clinical chorioamnionitis; HCA: histological chorioamnionitis; HCA & F: histological chorioamnionitis with funisitis mentioned separately. HCA and/or CCA: chorioamnionitis defined as positive when infants had histological or clinical chorioamnionitis; Microbiol: microbiological chorioamnionitis.

[c]Definition of chorioamnionitis: NoDes: no description; Des: clinical or histological description; Ref: defined according to cited article.

[d]Definition of ROP: ICROP: International Classification of Retinopathy of Prematurity; Ref: defined according to cited article; Treat: laser treatment of ROP; NA: no diagnostic criteria mentioned.

Supplementary Table 2. Newcastle-Ottawa Quality assessment of included studies.

First author, year	Perspective	Select.	Comp.	Outc.	Total	Reason for downgrade
Aden, 2013	IVH	4	0	2	6	No adjustment
Ahn, 2013	CA	4	1	3	8	Only adjusted for 1 factor
Alexander, 1998	CA	4	0	3	7	No adjustment
Arayici, 2004	CA	4	0	3	7	No adjustment
Austeng, 2010	CA	3	2	3	8	No CA definition
Babnik, 2006	CA-IVH	4	0	3	7	No adjustment
Barrera-Reyes, 2011	CA	4	0	2	6	No adjustment
Baumert, 2008	IVH	4	0	3	7	No adjustment
Been, 2009	CA	4	0	3	7	No adjustment
Bermick, 2016	IVH	3	2	3	8	No CA definition
Bordigato, 2010	CA	4	0	3	7	No adjustment
Botet, 2010	CA	4	1	3	8	Only adjusted for 1 factor
Bry, 2015	CA	4	0	2	6	No adjustment
Dalton, 2015	IVH	3	0	3	6	No CA definition
Dempsey, 2005	CA	4	0	3	7	No adjustment
Dexter, 2000	CA	4	0	2	6	Loss to follow up, no adjustment
Ecevit, 2014	CA	4	0	2	6	No IVH definition, no adjustment
Elimian, 2000	CA	4	2	3	9	
Erdemir, 2013	CA	4	0	2	6	No IVH definition, no adjustment
Fung, 2003	CA	4	0	2	6	No IVH definition, no adjustment
Gagliardi, 2014	CA	4	2	3	9	
Garcia-Munoz Rodrigo, 2014	CA	4	2	3	9	
Gawade, 2013	IVH	3	0	3	6	No CA definition, no adjustment
Gonzalez-Luis, 2002	CA	4	0	3	7	No adjustment
Gray, 1997	CA	4	0	3	7	
Hendson, 2011	CA	4	0	3	7	No adjustment
Hitti, 2001	CA	4	0	3	7	No adjustment
Holcroft, 2003	IVH	2	0	3	5	No IVH definition, no adjustment
Kallankari, 2010	IVH	4	0	3	7	No adjustment
Kaulkola,2006	IVH	4	0	3	7	No adjustment
Kidokoro	IVH	4	1	3	8	Only adjusted for 1 factor
Kim, 2015	CA	4	0	3	7	No adjustment
Kirchner, 2007	CA	4	0	3	7	No adjustment
Klebermans-Schrehof	IVH	4	0	3	7	No adjustment
Kosuge, 2000	CA	4	0	2	6	No IVH definition, no adjustment
Lau, 2005	CA	4	0	3	7	No adjustment
Lee Hyun Ju, 2011	CA	4	0	3	7	No adjustment

First author, year	Perspective	Select.	Comp.	Outc.	Total	Reason for downgrade
Lee Ju Young, 2010	IVH	4	1	3	8	Only adjusted for 1 factor
Lim, 2011	IVH	4	2	3	9	
Linder, 2003	IVH	4	2	3	9	
Liu, 2014	CA	4	0	3	7	No adjustment
Logan, 2013	IVH	4	0	3	7	No adjustment
Lu, 2016	IVH	4	0	3	7	No adjustment
Mehta, 2006	CA-IVH	4	0	3	7	No adjustment
Mestan, 2010	CA	4	0	3	7	No adjustment
Miyazaki, 2016	CA	4	2	3	9	
Morales, 1987	CA	4	0	3	7	No adjustment
Mu, 2008	CA	4	1	3	8	Only adjusted for 1 factor
Nasef, 2013	CA	4	0	3	7	No adjustment
Ogunyemi, 2003	CA	4	0	3	7	No adjustment
Oh, 2015	IVH	4	2	3	9	
Oh, 2018	CA	4	2	3	9	
Ohyama, 2002	CA	4	0	2	6	No IVH definition, no adjustment
Osmanagaoglu, 2005	CA	4	0	3	7	No adjustment
Pappas, 2014	CA	4	0	2	6	No IVH definition, no adjustment
Perrone, 2012	CA	4	0	3	7	No IVH definition
Polam, 2005	CA	4	0	2	6	Loss to follow up, no adjustment
Poralla, 2012	IVH	4	0	2	6	No IVH definition
Richardson, 2006	CA	4	0	2	6	No IVH definition, no adjustment
Rocha, 2006 & Rocha, 2007	CA	4	2	3	9	
Rodríguez-Trujillo, 2016	CA	4	0	3	7	No adjustment
Rong, 2012	IVH	4	2	2	8	No CA definition
Ryckman, 2011	IVH	3	0	3	6	No CA definition, no adjustment
Salas, 2013	CA	4	0	3	7	No adjustment
Sarkar, 2005	CA-IVH	4	0	3	7	No adjustment
Sato, 2011	CA	4	1	2	7	No IVH definition, only adjusted for 1 factor
Seliga-Siwecka, 2013	CA	4	2	3	9	
Shankaran, 2014	IVH	4	2	2	8	
Smit, 2015	CA	4	0	3	7	No adjustment
Soraisham, 2009	CA	4	2	3	9	
Soraisham, 2013	CA	4	0	2	6	Loss to follow up, no adjustment
Suarez, 2001	IVH	4	0	3	7	No adjustment
Suppiej, 2009	CA	4	0	2	6	Loss to follow up, no adjustment
Trevisanuto, 2010	CA	4	1	3	8	Only adjusted for 1 factor
Tsiartas, 2013	CA	4	0	3	7	No adjustment
Vaihinger, 2013	IVH	4	2	3	9	
van Vliet, 2012	CA	4	0	2	6	No adjustment
Vergani, 2004	IVH	4	0	3	7	No adjustment
Watterberg, 1999	CA	4	0	3	7	No adjustment

First author, year	Perspective	Select.	Comp.	Outc.	Total	Reason for downgrade
Wirbelauer, 2011	CA	4	0	3	7	No adjustment
Xu, 2012	CA	4	0	3	7	No adjustment
Yamada, 2015	CA	4	0	3	7	No adjustment
Yanowitz, 2006	CA	4	0	3	7	No adjustment
Yoon, 1995	CA	4	0	3	7	No adjustment
Zanardo, 2008	CA-IVH	4	0	3	7	No adjustment

Select.: selection; Comp.: comparability; Outc.: outcome; CA: chorioamnionitis; IVH: intraventricular hemorrhage

Supplementary Table 3. Meta-analysis of CA and all grades IVH, of studies with unadjusted and adjusted data.

CA type	Study	Crude OR (95% CI)	*p*	Adjusted OR (95% CI)	*p*	Confounders included in analysis
**Clinical**	Suarez 2001	4.18 (2.16-8.09)	<0.001	3.30 (1.52-1.12)	0.003	GA, BW, vaginal delivery, sepsis, RDS, death, tocolysis with magnesium, tocolysis with indomethacin and combined tocolysis with indomethacin and magnesium
**Histological**	Elimian 2000	1.56 (1.22-2.01)	<0.001	1.03 (0.77-1.37)	0.839	"Potential Confounders", not specified further
	Lu 2016	2.92 (1.29-6.57)	0.010	1.56 (1.13-2.15)	0.007	GA, BW, asphyxia resuscitation
	Miyazaki 2016	1.74 (1.46-2.09)	<0.001	1.11 (0.90-1.37)	0.33	GA, BW Maternal age, parity, diabetes, preeclampsia, preterm rupture of membranes, non-reassuring fetal status, antenatal corticosteroids, mode of delivery, gestational age of delivery, birth weight, SGA and sex of the infant
	Mu 2008	1.22 (0.51-2.95)	0.651	0.68 (0.25-1.86)	0.452	GA
	Oh 2015	NA	<0.001	0.72 (0.23-2.18)	0.555	GA and BW
	Rocha 2006	1.83 (1.10-3.03)	0.019	1.09 (0.51-2.34)	0.825	GA and BW
	Sato 2011	2.15 (1.27-3.65)	0.004	1.10 (0.90-1.34)	0.352	GA
	Bermick 2016	2.06 (1.18-3.61)	0.012	2.40 (1.19-4.85)	0.015	Male gender, pregnancy induced hypertension
	**Histological**	1.75 (1.54-1.99)	<0.001	1.17 (1.00-1.38)	0.056	
	**Overall**	1.80 (1.59-2.05)	<0.001	1.25 (1.02-1.53)	0.034	

CA: chorioamnionitis; OR: odds ratio; CI: confidence interval; GA: gestational age; BW: birth weight; RDS: respiratory distress syndrome; SGA: small for gestational age.

Supplementary Table 4. Meta-analysis of CA and grades 3-4 IVH. of studies with unadjusted and adjusted data.

CA type	Study	Crude OR (95% CI)	*p*	Adjusted OR (95% CI)	*p*	Confounders included in analysis
Clinical	Austeng 2010	NA		1.4 (0.71-2.75)	0.329	GA and BW
	Gagliardi 2014	3.65 (2.91-4.58)	<0.001	1.54 (1.14-2.08)	0.005	GA. antenatal steroids. gender. multiple pregnancies. inborn/outborn. and mode of delivery.
	Garcia-Munoz Rodrigo 2014	1.85 (1.57-2.19)	<0.001	0.89 (0.69-1.13)	0.323	GA. birth weight. sex. maternal hypertension. antenatal steroids. maternal antibiotics. multiplicity. type of delivery. necessity of advanced CPR. and CRIB
	Kidokoro 2014	NA	<0.001	0.51 (0.11-2.43)	0.398	Centre
	Soraisham 2009	2.09 (1.64-2.68)	<0.001	1.62 (1.17-2.24)	0.004	GA. BW. vaginal delivery. antenatal steroid and maternal hypertension. and Apgar score at 5 minutes. but not SNAP-II in the model.
	**Clinical**	2.41 (1.63-3.56)	<0.001	1.26 (0.93-1.69)	0.132	
Histological	Ahn 2012	9.94 (1.14-86.45)	0.037	0.47 (0.07-3.08)	0.431	GA
	Elimian 2000	1.78 (1.26-2.52)	0.001	1.2 (0.82-1.75)	0.346	"Potential Confounders". not specified further
	Mu 2008	1.06 (0.4-2.79)	0.905	0.54 (0.18-1.61)	0.265	GA
	Rocha 2006	2.33 (1.2-4.52)	0.012	0.94 (0.39-2.27)	0.891	GA and BW
	Seliga-Siwecka 2013	1.29 (0.85-1.96)	0.227	1.29 (0.85-1.96)	0.232	GA. PROM. antenatal steroids. mode of delivery
	**Histological**	1.7 (1.12-2.58)	0.013	1.06 (0.73-1.54)	0.758	
**Overall**		2.05 (1.54-2.72)	<0.001	1.22 (1.04-1.43)	0.013	

CA: chorioamnionitis; IVH: intraventricular hemorrhage; OR: odds ratio; CI: confidence interval; GA: gestational age; BW: birth weight; CPR: cardiopulmonary resuscitation; CRIB: clinical risk index; SNAP: Score for Neonatal Acute Physiology; PROM: premature rupture of membranes.

1.2 Supplementary Figures

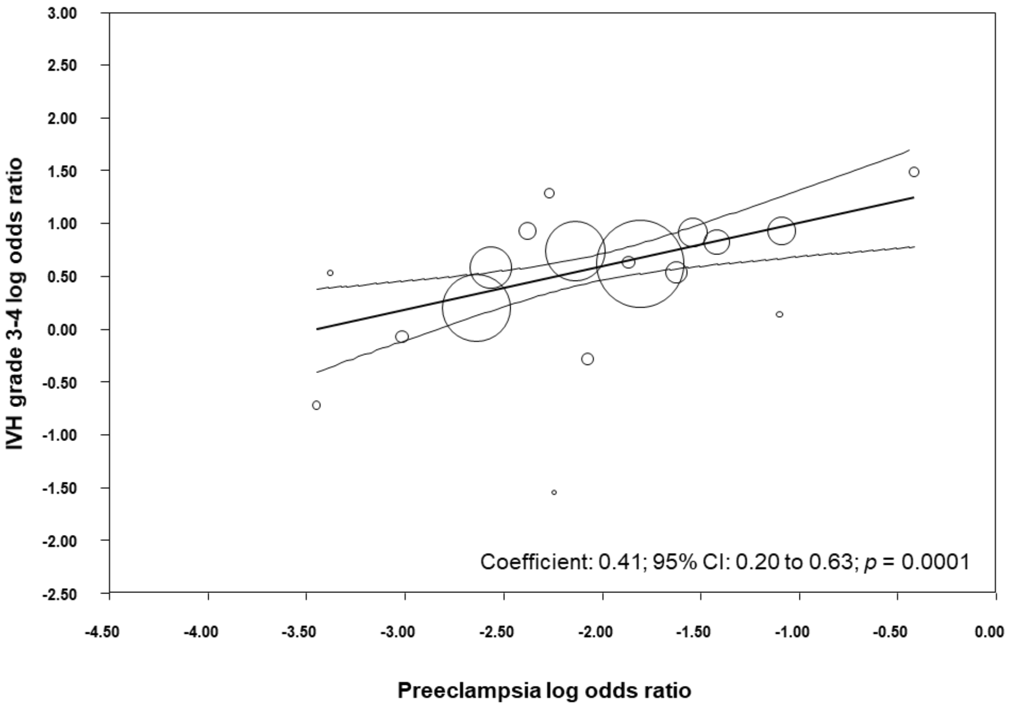

Supplementary Figure 1. Meta-regression of risk of intraventricular hemorrhage (IVH) grades 3-4 in the chorioamnionitis (CA) group and risk of preeclampsia in the CA group.

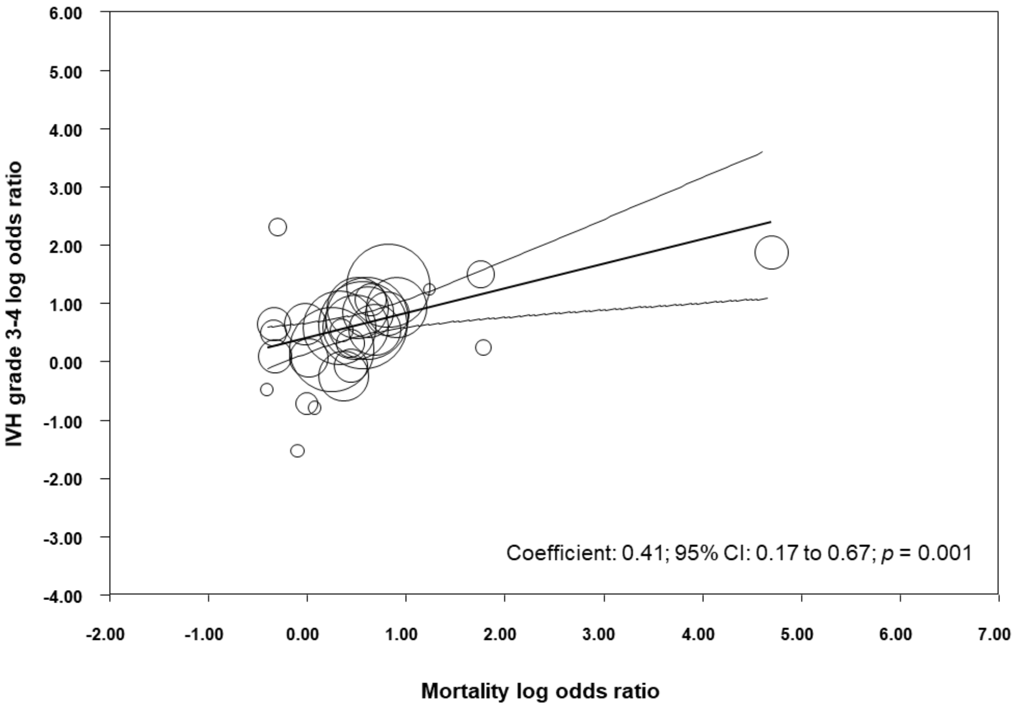

Supplementary Figure 2. Meta-regression of risk of intraventricular hemorrhage (IVH) grades 3-4 in the chorioamnionitis (CA) group and risk of mortality in the CA group.

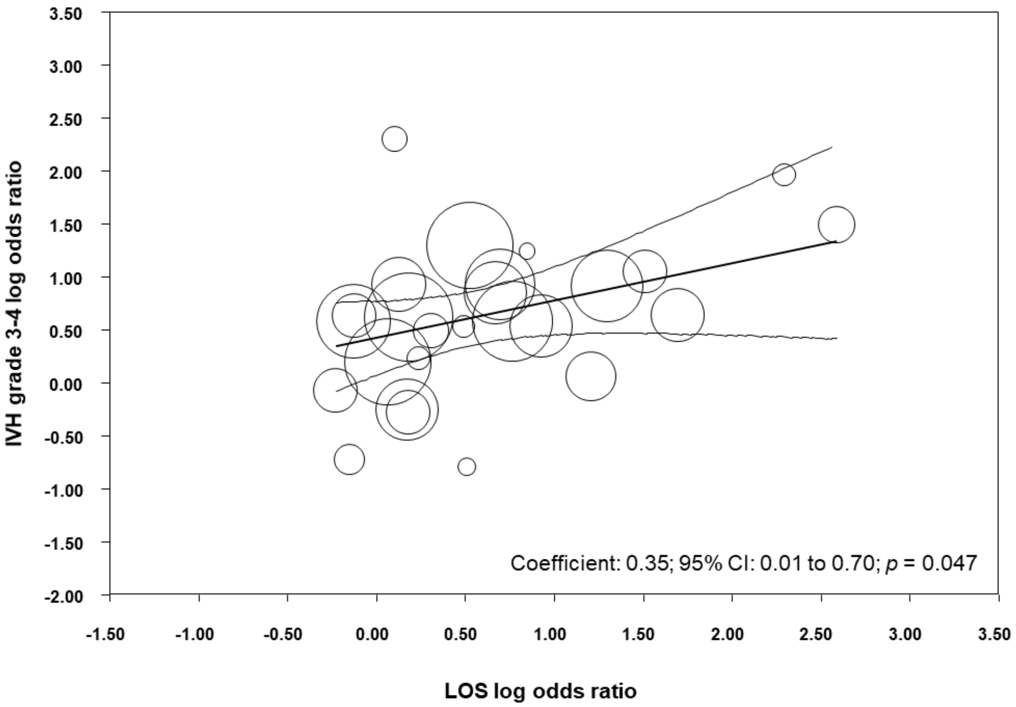

Supplementary Figure 3. Meta-regression of risk of intraventricular hemorrhage (IVH) grades 3-4 in the chorioamnionitis (CA) group and risk of late onset sepsis (LOS) in the CA group.

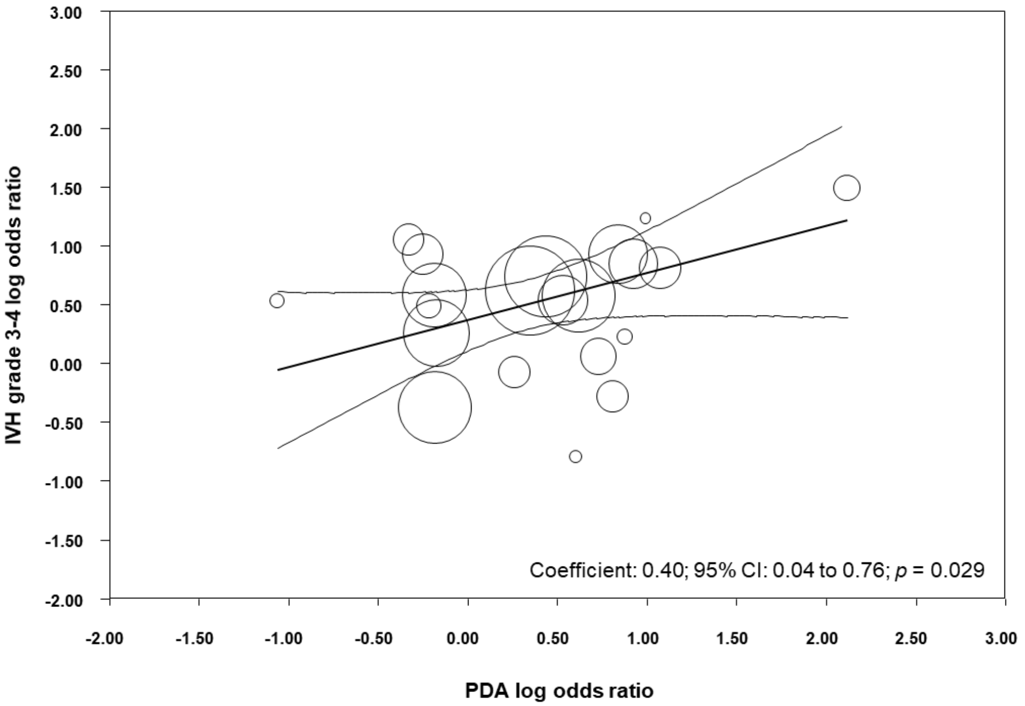

Supplementary Figure 4. Meta-regression of risk of intraventricular hemorrhage (IVH) grades 3-4 in the chorioamnionitis (CA) group and risk of paten ductus arteriosus (PDA) in the CA group.

PRISMA 2009 Checklist

Section/topic	#	Checklist item	Reported on page #
**TITLE**			
Title	1	Identify the report as a systematic review, meta-analysis, or both.	1
**ABSTRACT**			
Structured summary	2	Provide a structured summary including, as applicable: background; objectives; data sources; study eligibility criteria, participants, and interventions; study appraisal and synthesis methods; results; limitations; conclusions and implications of key findings; systematic review registration number.	1
**INTRODUCTION**			
Rationale	3	Describe the rationale for the review in the context of what is already known.	2
Objectives	4	Provide an explicit statement of questions being addressed with reference to participants, interventions, comparisons, outcomes, and study design (PICOS).	2
**METHODS**			
Protocol and registration	5	Indicate if a review protocol exists, if and where it can be accessed (e.g., Web address), and, if available, provide registration information including registration number.	2
Eligibility criteria	6	Specify study characteristics (e.g., PICOS, length of follow-up) and report characteristics (e.g., years considered, language, publication status) used as criteria for eligibility, giving rationale.	2
Information sources	7	Describe all information sources (e.g., databases with dates of coverage, contact with study authors to identify additional studies) in the search and date last searched.	2
Search	8	Present full electronic search strategy for at least one database, including any limits used, such that it could be repeated.	2
Study selection	9	State the process for selecting studies (i.e., screening, eligibility, included in systematic review, and, if applicable, included in the meta-analysis).	2,3
Data collection process	10	Describe method of data extraction from reports (e.g., piloted forms, independently, in duplicate) and any processes for obtaining and confirming data from investigators.	3
Data items	11	List and define all variables for which data were sought (e.g., PICOS, funding sources) and any assumptions and simplifications made.	3
Risk of bias in individual studies	12	Describe methods used for assessing risk of bias of individual studies (including specification of whether this was done at the study or outcome level), and how this information is to be used in any data synthesis.	3
Summary measures	13	State the principal summary measures (e.g., risk ratio, difference in means).	3
Synthesis of results	14	Describe the methods of handling data and combining results of studies, if done, including measures of consistency (e.g., I^2) for each meta-analysis.	3

PRISMA 2009 Checklist

Section/topic	#	Checklist item	Reported on page #
Risk of bias across studies	15	Specify any assessment of risk of bias that may affect the cumulative evidence (e.g., publication bias, selective reporting within studies).	3
Additional analyses	16	Describe methods of additional analyses (e.g., sensitivity or subgroup analyses, meta-regression), if done, indicating which were pre-specified.	3
**RESULTS**			
Study selection	17	Give numbers of studies screened, assessed for eligibility, and included in the review, with reasons for exclusions at each stage, ideally with a flow diagram.	3,4
Study characteristics	18	For each study, present characteristics for which data were extracted (e.g., study size, PICOS, follow-up period) and provide the citations.	3, Suppl. Table 1
Risk of bias within studies	19	Present data on risk of bias of each study and, if available, any outcome level assessment (see item 12).	4, Suppl. Table 2
Results of individual studies	20	For all outcomes considered (benefits or harms), present, for each study: (a) simple summary data for each intervention group (b) effect estimates and confidence intervals, ideally with a forest plot.	4,5
Synthesis of results	21	Present results of each meta-analysis done, including confidence intervals and measures of consistency.	4-12
Risk of bias across studies	22	Present results of any assessment of risk of bias across studies (see Item 15).	4,9
Additional analysis	23	Give results of additional analyses, if done (e.g., sensitivity or subgroup analyses, meta-regression [see Item 16]).	10-12
**DISCUSSION**			
Summary of evidence	24	Summarize the main findings including the strength of evidence for each main outcome; consider their relevance to key groups (e.g., healthcare providers, users, and policy makers).	7
Limitations	25	Discuss limitations at study and outcome level (e.g., risk of bias), and at review-level (e.g., incomplete retrieval of identified research, reporting bias).	13
Conclusions	26	Provide a general interpretation of the results in the context of other evidence, and implications for future research.	13
**FUNDING**			
Funding	27	Describe sources of funding for the systematic review and other support (e.g., supply of data); role of funders for the systematic review.	17

From: Moher D, Liberati A, Tetzlaff J, Altman DG, The PRISMA Group (2009). Preferred Reporting Items for Systematic Reviews and Meta-Analyses: The PRISMA Statement. PLoS Med 6(7): e1000097. doi:10.1371/journal.pmed1000097
